# Evaluating Behavioural Interventions for Oropharyngeal Dysphagia in Adults: A Systematic Review and Meta-Analysis of Swallowing Manoeuvres, Exercises, and Postural Techniques †

**DOI:** 10.3390/jcm14207180

**Published:** 2025-10-11

**Authors:** Silvia Adzimová, Renée Speyer, Reinie Cordier, Catriona Windsor, Žofia Korim, Miroslav Tedla

**Affiliations:** 1 Department of Otolaryngology, Head and Neck Surgery, Central Military Hospital SNP Ružomberok—Faculty Hospital, 03426 Ružomberok, Slovakia; silviaadzimova@gmail.com; 2 Department of Otolaryngology, Head and Neck Surgery, Comenius University, University Hospital, 85107 Bratislava, Slovakia; zofia.frajkova@gmail.com (Ž.K.); miro.tedla@gmail.com (M.T.); 3 Discipline of Speech and Language Therapy, School of Health Sciences, College of Medicine, Nursing & Health Sciences, University of Galway, H91 TK33 Galway, Ireland; 4 Curtin School of Allied Health, Faculty of Health Sciences, Curtin University, Perth, WA 6102, Australia; reinie.cordier@curtin.edu.au; 5 Department of Social Work, Education and Community Wellbeing, Northumbria University, Newcastle upon Tyne NE1 8ST, UK; 6 Department of Health & Rehabilitation Sciences, Faculty of Health Sciences, University of Cape Town, Cape Town 7700, South Africa; 7 Department of Special Needs Education, University of Oslo, 0318 Oslo, Norway; catriona.windsor@isp.uio.no; 8 Department of Neurology, Faculty Hospital Trnava, 91775 Trnava, Slovakia; 9 Department of Communication Disorders, Faculty of Education, Comenius University Bratislava, 83102 Bratislava, Slovakia; 10 Institute of Cancer and Genomic Sciences, University of Birmingham, Birmingham B15 2SQ, UK

**Keywords:** swallowing, swallowing disorders, deglutition disorders, behavioural interventions, compensation, rehabilitation, postural strategies, swallowing manoeuvres, swallowing exercises

## Abstract

**Background/Objectives**: To assess the effectiveness of the most commonly used swallowing manoeuvres, exercises, and postural strategies as standalone interventions in the behavioural management of oropharyngeal dysphagia in adults. **Methods**: Systematic searches of two electronic databases, Embase and PubMed, were conducted in accordance with PRISMA guidelines to identify studies with comparison groups, including (pseudo) randomised controlled trials, comparative studies with concurrent controls, and within-subject or crossover study designs. The methodological quality of the included studies was assessed using the standard quality assessment tool (QualSyst). **Results**: Seventeen studies met the eligibility criteria, evaluating the effects of chin tuck, effortful swallow, the Mendelsohn manoeuvre, the modified jaw opening exercise, the volitional laryngeal vestibule closure manoeuvre, and the Shaker exercise. Most studies reported positive treatment outcomes, supporting the benefits of both compensatory and rehabilitative interventions across diverse populations, mainly derived from mixed groups and individuals post stroke. However, due to the limited number and significant heterogeneity of studies, a meta-analysis was only performed for the chin tuck, which showed a moderate positive effect. Overall, the evidence is preliminary and should be interpreted with caution. **Conclusions**: While current findings support the benefits of standalone behavioural interventions in oropharyngeal dysphagia, future research should focus on high-quality study designs with larger populations. Such studies need to consider variability in patient characteristics, intervention protocols, and outcome measures, with the use of advanced statistical methods enabling more definitive conclusions about the effectiveness of these interventions.

## 1. Introduction

Swallowing is a complex physiological process involving multiple structures responsible for transferring a bolus from the oral cavity to the stomach. Dysphagia denotes swallowing disorders, which may impact the oropharyngeal phase (oropharyngeal dysphagia [OD]) and/or the esophageal phase (esophageal dysphagia). The definition of dysphagia was established through an international expert consensus using the Delphi technique [1], and describes dysphagia as “…*a symptom or a collection of symptoms that may result from one or more underlying anatomical abnormalities or impairments in the cognitive, sensory, and motor functions involved in transferring substances, including food and liquids, from the mouth (or nasal cavity) to the stomach*” [2] [p. 5; Table 1]. The condition can result in decreased swallowing efficiency and safety, failure to sustain adequate hydration and nutrition, and increased risks such as choking, aspiration, and diminished quality of life [2]. Safety pertains to the capacity to safeguard the airway from liquids and food entering the lungs (aspiration), while efficiency concerns the coordination and timing of swallowing muscles that facilitate the effective transport of food and liquids, ensuring that no bolus residue remains in the oral cavity, pharynx, or larynx [3].

The prevalence and aetiologies of dysphagia exhibit significant variability across different age demographics and related health challenges. Gastroesophageal or immunological conditions are more frequently observed in middle-aged individuals, whereas neurological or oncological causes predominantly affect the elderly [4]. OD affects 2.3–16.0% of the general population [5] and is more prevalent in specific groups: for example, 8.1–80.0% in stroke, 11.0–81.0% in Parkinson’s disease, 27.0–30.0% in traumatic brain injury, 91.7% in community-acquired pneumonia [6], 32.8% in the elderly [7], and 21.9–69.5% in antipsychotic users [8]. Furthermore, the prevalence of OD differs among various healthcare settings: 36.5% of people in hospitals, 42.5% in rehabilitation centres, and 50.2% in nursing homes are impacted [9].

The management of dysphagia may encompass surgical, pharmacological, and/or behavioural interventions, employed either independently or in conjunction [10,11,12,13,14]. Surgical interventions aim to address structural abnormalities or functional deficits, such as dilation of the upper esophageal sphincter (UES) [15,16], whereas pharmacological procedures concentrate on managing symptoms and underlying conditions. For example, prokinetics are used to enhance gastrointestinal motility, and antimicrobial therapies are employed to treat infections [17,18]. Behavioural interventions, conversely, are concentrated on altering swallowing behaviour through compensatory and/or rehabilitative treatment strategies [13,19].

Compensatory strategies offer immediate yet temporary enhancements in swallowing function by modifying bolus timing or pathway to diminish the risk of aspiration, thereby ensuring safer oral intake while the underlying swallow deficit persists [20]. These strategies encompass postural modifications, selected swallowing manoeuvres, bolus modification, sensory enhancements, as well as adjustments to mealtimes and the dining environment [12,21,22]. Rehabilitative strategies aim for the long-term restoration of swallowing function by focusing on muscle performance (such as strength, mobility, timing, and coordination) and central neural plasticity [10,23,24]. Example strategies encompass exercises such as lip or tongue strengthening activities, the Masako manoeuvre, the Mendelsohn manoeuvre, supraglottic and super-supraglottic swallowing techniques, the effortful swallow, the Shaker exercise [25,26], and neuromuscular electrical stimulation (NMES) [27].

Positional and postural techniques [28,29] involve modifications in body positioning and alignment of the head or neck prior to the commencement of the pharyngeal phase of swallowing, with these adjustments maintained until the swallowing process is complete [30]. The goal of these strategies is to modify the bolus trajectory within the oral cavity and pharynx [22,31], shorten both oral and pharyngeal transit durations [32], minimise residual matter following swallowing, and either reduce or completely eliminate the risk of aspiration [29,33,34,35,36]. A noteworthy benefit of these interventions is their relative simplicity, necessitating minimal effort from the patient to learn and implement.

Swallowing manoeuvres pertain to specific strategies involving voluntary movements of the oral, pharyngeal, or laryngeal structures, which are executed prior to or during the pharyngeal phase of swallowing [37]. Their application entails modifying particular elements of swallowing physiology, including bolus propulsion and airway protection [37,38]. Typically, swallowing manoeuvres presuppose the patient’s capability to adhere to detailed instructions. Consequently, patients must possess sufficient cognitive functions (e.g., the ability to follow and execute multiple directives), intact praxis, and the motivation to participate in autonomous practice and intensive training [39,40]. Swallowing manoeuvres may serve a compensatory function by immediately modifying the mechanics of swallowing to mitigate the risk of aspiration, thereby promoting safer swallowing during use. Concurrently, when practised consistently over an extended period, these manoeuvres can have a rehabilitative effect by strengthening specific muscles and improving neural control of the swallowing process, potentially resulting in lasting functional enhancements even without active execution of the manoeuvres [41,42,43]. Furthermore, several oromotor and neuromuscular exercises have been documented in the scholarly literature (e.g., [13,44,45,46]), focusing on improving bolus control and the oral phase of swallowing, in addition to strengthening muscles and enhancing coordination to optimise pharyngeal and laryngeal function. Supplementary Table S1 provides examples of the most frequently employed swallowing manoeuvres, exercises, and postural strategies for the treatment of OD in adults.

Over the past few decades, numerous studies have documented the effects of behavioural interventions in the field of OD. To synthesise findings, various systematic reviews have examined the impact of specific behavioural interventions, such as the effortful swallow manoeuvre [38], the chin tuck against resistance [47], or head and neck postures [35]. Other reviews have focused on particular patient populations, including Parkinson’s disease [48], acute and critical care [49], head and neck cancer [50,51], and stroke [52]. To date, only two systematic reviews have evaluated the effects of behavioural interventions in people with OD without restriction to a specific intervention or patient population [13,46]. Nonetheless, the first review [46], conducted in 2010, did not incorporate more recent studies, whereas the latest review from 2022 included solely randomised controlled trials [13].

The objective of this systematic review was to ascertain the effectiveness of the most frequently utilised swallowing manoeuvres, exercises, and postural strategies, as standalone interventions in the behavioural management of OD in adults, without regard to the underlying medical diagnosis. The focus was on isolating the effects of individual intervention components (i.e., standalone intervention), thereby excluding combined therapies (i.e., therapies involving multiple postural strategies and/or swallowing manoeuvres or exercises) where the outcomes of a specific strategy or manoeuvre could not be distinguished from the overall therapeutic effects.

## 2. Methods

The methodology and reporting of this systematic review adhered to the guidelines outlined in the “Preferred Reporting Items for Systematic Reviews and Meta-Analyses” (PRISMA) statement, its checklist, and PRISMA-S, the extension for reporting literature searches (Supplementary Files S1–S3). These guidelines and standards are designed to enhance the quality and transparency of reporting in systematic reviews [53,54].

### 2.1. Information Sources

On 5 May 2025, systematic searches were conducted across two electronic databases: Embase (Ovid) and PubMed. Publication dates ranged from 1902 to 2025 and from the late 1700s to 2025, respectively. Additional searches involved reviewing the reference lists of eligible articles.

### 2.2. Search Strategies

Electronic search strategies were conducted across two databases using both subheadings (such as Medical Subject Headings [MeSHs] and Thesaurus terms) and free-text terms. No language or other restrictions were applied to any of the searches. The full electronic search strategies are detailed in Table 1.

### 2.3. Eligibility Criteria

The following criteria were used to select studies: (1) participants were adults diagnosed with OD confirmed by instrumental assessment (i.e., FEES [Fiberoptic Endoscopic Evaluation of Swallowing] and/or VFSS [Video Fluoroscopic Swallow Study]); (2) interventions targeted reducing swallowing difficulties through one or more of the most commonly used postural strategies, swallowing manoeuvres, and/or exercises (e.g., chin tuck [chin down], head flexion [neck flexion], chin up [head back or head extension], effortful swallow, head tilt, head turn [head rotation], Masako manoeuvre [tongue hold], Mendelsohn manoeuvre, side lying, Shaker exercise [head lift], super supraglottic swallow, and supraglottic swallow); (3) studies reported data before and after the intervention; (4) studies included a comparison group, such as (pseudo)randomised controlled trials, comparative studies with concurrent controls, and within-subject or crossover study designs [55]; (5) the minimum number of participants was more than five subjects per group; (6) only original studies were included; and (7) studies were published in English.

Studies focusing on healthy older adults with sarcopenia and/or muscle wasting (presbyphagia), or those evaluating swallowing training in healthy individuals, were ineligible. Interventions aimed at esophageal dysphagia or describing proactive treatments (e.g., in patients with head and neck cancer before radiation) were also excluded, along with studies reporting on combined interventions (e.g., postural strategies and/or swallowing manoeuvres combined with diet modification or thermal-tactile stimulation). Additionally, papers and reports that were not original research (e.g., conference abstracts, dissertations, and reviews) were not considered eligible.

### 2.4. Systematic Review

*Study selection:* Two independent abstract reviewers conducted a stepwise assessment of eligibility. First, titles and abstracts were screened for eligibility using predefined criteria. After excluding reports that did not meet the inclusion criteria, the full-text articles of the remaining records were retrieved for further review. Both reviewers evaluated all full-text articles for eligibility. Disagreements about article eligibility were resolved by consensus. If disagreements persisted, a third reviewer was consulted to make the final decision on the studies’ eligibility.

*Methodological Quality Assessment:* The methodological quality of the included studies was evaluated using the standard quality assessment tool (QualSyst) as outlined by Kmet et al. [56], in conjunction with the National Health and Medical Research Council (NHMRC) Evidence Hierarchy levels of evidence [55].

The Qualsyst critical appraisal tool offers a systematic, reproducible, and quantitative method for assessing the methodological quality of studies across a wide range of study designs. QualSyst ensures that studies selected for the systematic review meet a minimum quality standard. A total score based on 14 QualSyst criteria is converted into an overall quality percentage score: dividing the total score by the number of applicable items and multiplying by 100. An overall percentage score of 80% or higher signifies strong methodological quality; scores between 70% and 79% indicate good quality; scores from 50% to 69% denote adequate quality; and scores below 50% suggest poor methodological quality.

The NHMRC guidelines were followed to ensure the systematic review adhered to evidence-based practices and maintained high methodological standards. These guidelines provide a framework that categorises studies into different levels of evidence based on their design quality, enabling practitioners to evaluate the reliability and relevance of research outcomes. The NHMRC describes the levels of evidence as follows: Level I includes systematic reviews of randomised controlled trials; Level II is made up of randomised controlled trials; Level III-1 involves pseudo-randomised controlled trials; Level III-2 covers comparative studies with concurrent controls; Level III-3 pertains to comparative studies with historical control, two or more single-arm studies, or interrupted time series without a control group; and Level IV consists of case series with either post-test or pre-test/post-test outcomes. Only studies at NHMRC levels II and III were deemed eligible.

*Data Extraction Process:* A data extraction form was created to gather and organise data from the included studies, enabling systematic comparisons. Data were extracted under the following categories: NHMRC study design and QualSyst score; OD (definition, diagnostic measures and eligibility criteria); study population (medical diagnoses and sample sizes); group demographics (age, gender and detailed medical diagnoses); intervention goals, materials and procedures; reported outcome measures; and therapy outcomes.

*Data Items, Risk of Bias, and Synthesis of Results:* The risk of bias was assessed at the individual study level using the QualSyst critical appraisal tool. Throughout the review process, the risk of process bias was minimised by ensuring full overlap between two independent reviewers during record and article selection, methodological quality assessment, and data extraction. Discrepancies were resolved through consensus. In cases where both reviewers could not reach consensus, a third reviewer was called in to facilitate a resolution. Extracted information was categorised as described above. Intervention outcomes were evaluated based on effect sizes and statistical significance. However, due to variability in patient characteristics, intervention designs and outcome measures in the included studies, a meta-analysis was only feasible for selected studies based on the chin tuck manoeuvre [57].

### 2.5. Meta-Analysis

Data were extracted from relevant studies to compare effect sizes for pre-post outcome measures of OD, as well as the mean differences in outcomes from pre- to post-intervention across different types of standalone behavioural interventions (i.e., swallowing manoeuvres, exercises, and postural strategies). Depending on the study design (e.g., crossover designs), pre- and post-intervention assessments were interpreted as evaluations of the same participants under two different conditions—for example, performing a standard swallow in a neutral head position (pre-intervention) versus executing a swallowing manoeuvre or adopting a postural strategy (post-intervention).

To reduce heterogeneity between studies, only outcome measures derived from instrumental assessments and from clinical non-instrumental assessments were considered eligible for inclusion in the meta-analyses. When both types of data were reported, outcomes from instrumental assessments were prioritised. Measures such as oral intake scales, screening tools, and patient-reported outcomes were excluded from the meta-analyses. In some cases, outcomes other than those designated as primary by the original study authors were selected if doing so contributed to reducing heterogeneity across studies.

To facilitate the comparison of effect sizes, group means, standard deviations, and sample sizes for pre- and post-intervention measurements were entered into Comprehensive Meta-Analysis (Version 4.0) [58]. When only non-parametric data (e.g., medians, interquartile ranges) were reported, these were converted to parametric equivalents to allow inclusion in the meta-analysis. When necessary, data were missing or incomplete, study authors were contacted via email to request additional information.

Effect sizes were calculated using Comprehensive Meta-Analysis with a random-effects model, which was chosen to account for expected differences in true effects across studies due to variations in participant characteristics, intervention types, and outcome measures. Prediction intervals were used to assess heterogeneity in the meta-analysis [59]. Standardised mean differences were calculated using Hedges’ *g*, along with 95% confidence intervals. Effect sizes were interpreted according to Cohen’s conventions: Hedges’ *g* ≤ 0.2, no or a negligible effect, 0.2 < Hedges’ *g* ≤ 0.5, a small effect, 0.5 < Hedges’ *g* ≤ 0.8, a moderate effect, and Hedges’ *g* > 0.8, a large effect [60].

Forest plots were created to display effect sizes for outcome measures of OD after specific pre-post standalone behavioural interventions, where sufficient data were available. Publication bias was examined using Comprehensive Meta-Analysis software (version 4.0) with two statistical methods: Begg and Mazumdar’s rank correlation test and the Fail-safe N test. The Begg and Mazumdar test assesses the rank correlation between standardised effect sizes and their variances [61]. This analysis yields a Kendall’s tau coefficient along with a two-tailed *p*-value. A tau value of zero indicates no association, while values that differ from zero suggest a possible relationship between effect size and variance. If there is publication bias, studies with larger standard errors might show disproportionately larger effect sizes. A positive tau implies that smaller studies tend to report larger effects (meaning effect sizes decrease as standard error increases), whereas a negative tau suggests the opposite trend. The Fail-safe N test estimates how many hypothetical studies with null results (effect size = 0) would need to be added to the analysis to make the overall findings non-significant [62]. A small fail-safe N raises doubts about the robustness of the observed effect, indicating unpublished studies may influence it. Conversely, a large fail-safe N offers more confidence that the observed effect, even if inflated by publication bias, is unlikely to be purely due to bias or chance.

## 3. Results

### 3.1. Study Selection

**Figure 1:**
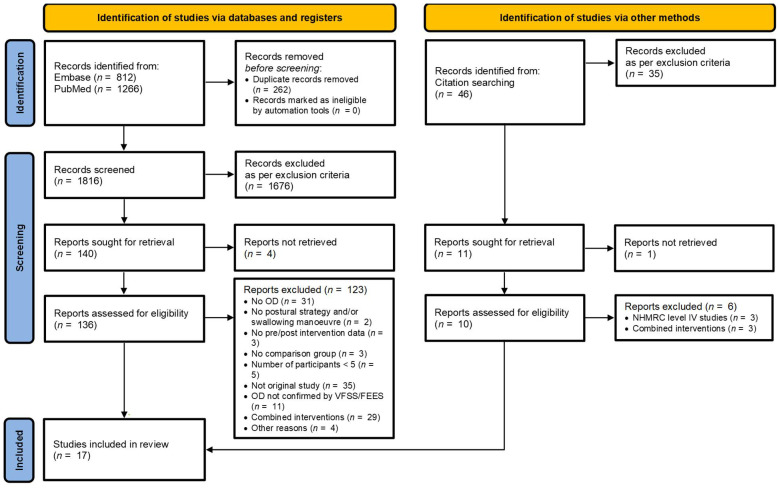
Flow diagram of the review process based on PRISMA [53].

The initial literature search was conducted using two electronic databases: Embase (*n* = 812) and PubMed (*n* = 1266), resulting in a total of 2078 studies. After removing 262 duplicate records with EndNote’s duplicate detection tool, 1816 unique titles and abstracts were screened by two independent reviewers based on the inclusion and exclusion criteria. The same two reviewers then independently evaluated the eligibility of 136 full-text articles (reports). A third reviewer was consulted to resolve any disagreements during this process. Ultimately, 13 studies met the inclusion criteria, and four additional studies were identified through reference checking of the included articles. A total of 17 studies were included in the final review. Figure 1 illustrates the PRISMA flow diagram summarising the article selection process [53].

### 3.2. Description of Studies

All 17 included studies are detailed in Table 2 and Table 3. Table 2 outlines the study characteristics (authors, publication year, country, study design, QualSyst score), OD details (terminology, assessment methods, diagnoses, eligibility criteria), and sample and group descriptions. Table 3 offers information on intervention specifics (goals, delivery and dosage), materials and procedures, outcome measures, and the primary treatment outcomes of the included studies.

*Participants* (Table 2): A total of 618 participants were included across the 17 studies, with sample sizes ranging from seven [63] to 97 [64]. However, one study showed minor inconsistencies in the reporting of participant numbers [65]. The reported mean ages across 14 studies [40,43,63,64,65,66,67,68,69,70,71,72,73,74] ranged from 43 years [74] to 77 years [68], while two studies [39,75] reported median ages of 74 and 76 years. One study [76] did not report on age demographics. The minimum and maximum ages across all studies ranged from 18 to 92 years. Overall, 15 of the included studies reporting on gender included a total of 349 (65%) men and 190 (25%) women. One additional study had minor inconsistencies in gender distribution [66], and two studies did not report the gender distribution of participants at all [67,76].

Most studies (*n* = 9) focused on mixed populations [64,67,69,70,71,73,74,75,76], including individuals with Parkinson’s disease or stroke [69], neurogenic dysphagia due to traumatic brain injury or stroke [74], and pharyngeal dysphagia associated with upper esophageal sphincter dysfunction, primarily resulting from stroke or post-radiation effects [75]. Six of these nine studies included mixed populations with a broad range of dysphagia aetiologies rather than focusing on specific diagnostic groups. An additional six studies specifically targeted stroke patients [39,40,43,65,66,72]. The two remaining studies focused on more specific populations, with one including patients with myasthenia gravis [63] and the other investigating two separate groups, consisting of individuals with stroke and general internal medicine patients [68].

The studies were published between 2002 and 2022 and conducted across eight countries: Brazil [69], Canada [68], Ireland [70], Japan [63,72,73,76], Republic of Korea [64,66,71], Spain [74], the UK [39], and the USA (*n* = 5) [40,43,65,67,75]. The most commonly used method for confirming OD was VFSS (*n* = 14) [40,43,64,65,66,67,68,70,71,72,73,74,75,76], while FEES was utilised in three studies (*n* = 3) [39,63,69].

*Outcome measures and instrumental methods* (Table 3): The included studies employed various outcome measures, each targeting different aspects of OD. VFSS was the most commonly used instrumental method for assessing intervention effects, documented in 13 studies [40,43,64,65,66,67,68,71,72,73,74,75,76]. Surface electromyography (sEMG) and high-resolution manometry (HRM) were the next most commonly reported instrumental methods, each used in two studies (HRM [63,70] and sEMG [39,69]).

In addition to various physiological and kinematic parameters, such as spatial, temporal, and distance-based measurements [40,43,64,67,68,71,72,73,74,75], several visuoperceptual evaluation measures were used to interpret VFSS or FEES recordings. These included the Penetration–Aspiration Scale (PAS), which was reported in multiple studies [40,67,68,73], the 8-point PAS (8PPAS) [64], and the Modified Barium Swallow Impairment Profile (MBSImP) [76]. Very few studies used functional outcome tools. Among those that did, the following were employed: the American Speech-Language-Hearing Association National Outcomes Measurement System (ASHA-NOMS) [66], the Dysphagia Outcome and Severity Scale (DOSS) [40], and the Functional Outcome Assessment of Swallowing (FOAMS) [75]. The limited use of these tools may be due to most studies involving single sessions, which limits repeated measurement and, consequently, the clinical translation of findings.

*Intervention agents* (Table 3): Interventions were delivered by various healthcare professionals, such as speech and language pathologists [68,70,75,76], physiotherapists (PTs) [66] and physiatrists [64]. One study employed a research technician as the intervention provider [73]. However, most studies did not specify who delivered the intervention [63,69], instead referring broadly to researchers and clinicians [39,40,43,65,67,74], or MJOE (Modified Jaw Opening Exercise) trainer [72]. The dosage and duration period of interventions varied considerably, depending on the specific intervention goal and training protocols of each specific behavioural strategy

*Behavioural intervention groups* (Table 3): Three studies assessed various interventions across two distinct populations with OD; these studies compared the (isometric/isotonic) Shaker exercise with proprioceptive neuromuscular facilitation (PNF) [66], the modified jaw opening exercise (MJOE) [72] and the Head-raising exercise [75]. Each was evaluated against a sham intervention. One study [65] examined the same intervention, volitional laryngeal vestibule closure (vLVC), across three patient groups, each receiving biofeedback under different circumstances: submental surface electromyography (ssEMG), VFFS, or a combination of both. The remaining 13 studies employed cross-over or retrospective designs to compare outcomes within the same population under different conditions. Two studies evaluated the effects of the Mendelsohn manoeuvre—one being a secondary analysis of the other [40,43]—as well as the effects of a no-treatment period. Three studies explored the effects of the effortful swallow with or without electromyography (EMG) biofeedback [39] or compared it to spontaneous swallowing [69,70]. Similarly, eight studies examined the effects of the chin-down posture compared to normal swallowing within the same population [63,64,67,68,71,73,74,76], with one study reporting separately on two distinct bolus administrations [68]. None of the studies reported long-term outcomes, which is consistent with the majority of research examining single-session interventions. Consequently, the studies may demonstrate improvements during the execution of a manoeuvre, but such improvements may not necessarily be sustained over an extended period.

**Table 2:** Study characteristics of studies on behavioural interventions for people with OD.

Author, Year **Country** **Study Design ^a^** **QualSyst Score ^b^**	**OD (Definition, Assessment)** **Medical Diagnosis** **Main Inclusion/Exclusion Criteria**	Sample (N) Groups ^c^ (*n*)	Group Descriptives (e.g., Age, Gender, Medical Diagnoses)				
**Archer et al., 2021** [39] **UK** **II** **Strong: 90%**	OD definition: difficulty with swallowing OD confirmed by FEES OD assessment: FOIS, PAS (FEES) Medical diagnosis (Group 1): adults after stroke **Inclusion, healthy:** volunteers, >18 years; able to eat/drink normal diet & fluids (determined by FOIS). **Exclusion, healthy:** history of dysphagia, stroke/other neurological/muscular illness/HNC/surgery (determined by self-report questions). **Inclusion, stroke:** ≤3 months post-stroke; SLT referral for dysphagia assessment & management; dysphagia confirmed FEES (including PAS); FOIS < 6; able to provide informed consent. **Exclusion, stroke:** FOIS score ≥ 6; previous history of dysphagia, stroke, neurological illness and/or HNC/surgery.	N = 13 (stroke patients) N = 17 (healthy controls) (crossover study design, random allocation): **- Condition 1:** ES **- Condition 2:** ES + biofeedback	**Demographics** Age *(yrs):* Median (IQR) Gender *(n_M_/n_F_)* FOIS: Median (IQR) PAS: Median (IQR)	Group Stroke * 74.5 (61.3–83.3) 9/5 4.0 (1.0–5.0) 7.5 (5.3–8.0)		**Group Healthy controls** 76 (74.5–81.5) 10/7 N/A N/A	
			* Prior to participant drop-out (*n* = 14): R MCA infarct (*5*); L MCA infarct (*3*); other (*6*)				
**Don Kim et al., 2015** [66] **Republic of Korea** **II** **Good: 71%**	OD definition: difficulty with chewing and swallowing food OD confirmed by VFSS Medical diagnosis (Group 1 & 2): adults after stroke **Inclusion:** symptoms of dysphagia for over 6 months prior to treatment; 24 points or higher on the Korean version of MMSE (MMSE-K); at least fair grade in manual muscle testing of the neck flexors; and voluntary agreement to participation in this study **Exclusion:** patients with any kind of heart disease, internal disease, or musculoskeletal disease, who would have had difficulty performing training	N = 26 (stroke patients, random allocation): - **Group 1** (*n* = 13): Shaker exercise - **Group 2** (*n* = 13): Proprioceptive neuromuscular facilitation (PNF)—based short neck flexion	**Demographics** Age *(yrs):* Mean ± SD After onset *(months):* Mean ± SD Gender *(n_M_/n_F_)* Side (R/L) * Inconsistency in reporting sample size	**Group 1** 63.6 ± 8.1 16.15 ± 3.1 7/8 * 7/6		**Group 2** 63.2 ± 10.2 15.6 ± 2.9 8/5 7/6	
**Forbes & Humbert, 2021** [67] **USA** **III-2** **Strong: 91%**	OD definition: N/R OD confirmed by VFSS Medical diagnosis: adults with various medical conditions **Inclusion:** deep penetration or aspiration on at least one bolus type during swallows in the neutral head position; ability to successfully perform the chin-down posture; at least one swallow in the chin-down posture and one swallow in a neutral head position with the same bolus type and delivery conditions. **Exclusion:** dementia (Mini-Mental State Examination score < 22), pregnancy, respiratory disease, or aphasia.	N = 15 (patients with OD of mixed aetiology, retrospective study). N = 62 paired swallows * categorized to: - **Condition 1** (*n* = 31 swallows): Chin-down - **Condition 2** (*n* = 31 swallows): Neutral head position * Real-time clinical judgments of bolus depth relative to swallow onset contributed to aberrant bolus flow and served as reasons for chin-down posture.	**Demographics** Age (*yrs*): Mean ± SD	**Group** 58.0 ± 18.0			
			Medical diagnoses (*n* = 15): cerebrovascular accident (*5*), unknown (*2*), coronary artery disease, recurrent urinary tract infection and chronic kidney disease (*1*); Karlengener’s syndrome, cancer, and base of tongue radiation (*1*); neurofibroma and transoral resection (*1*); open valve replacement complication by intubation resulting in injury (*1*); right laryngocele surgery (*1*); spinal surgery for cervical fusion (*1*); surgery for osteophytes (*1*); vocal cord paralysis (*1*).				
**Fraser & Steele, 2012** [68] **Canada** **III-1** **Strong: 82%**	OD definition: N/R OD/aspiration confirmed by VFSS OD assessment: swallowing assessment by SLP, VFSS Medical diagnosis (Groups 1 & 2): adults after stroke and general internal medicine (GIM) patients **Inclusion**: adult inpatient participants from acute care and rehabilitation units with aspiration during VFSS (thin liquid swallowing). **Exclusion:** no available chin down manoeuvre swallows on VFSS; history of head and neck cancer; tracheostomy tube in place; inability to follow instructions for chin down manoeuvre; physical limitations for sitting upright or flexing neck.	N = 42 - **Group 1** (*n* = 16): stroke - **Group 2** (*n* = 26): GIM patients N = 98 (VFSS swallows: de-identified and randomised for rating) categorised to: - **Condition 1** (*n* = 19)**:** Chin down + teaspoon administration - **Condition 2** (*n* = 30)**:** Chin down + cup drinking - **Condition 3** (*n* = 19)**:** Head neutral + teaspoon administration - **Condition 4** (*n* = 30)**:** Head neutral + cup drinking	**Demographics** Age (*yrs*): Mean (Range) After stroke onset: Gender *(n_M_/n_F_)*	**Group 1** 73 (49–87) 2 week(s)–18 month(s) 9/7		Group 2 * 77 (39–92) N/A 14/12	
			Medical diagnoses *: multiple sclerosis, chronic obstructive pulmonary disease (COPD), kidney disease, fractures, congestive heart failure, diabetes, sepsis, Wilson’s disease, and gastrointestinal disease. *NB*. Aetiology factor removed from analyses (as no significant group differences for frequency of airway invasion)				
**Gomes et al., 2020** [69] **Brazil** **III-2** **Good: 77%**	OD definition: presence of at least one of the subsequent outcomes, namely presence of posterior oral spillage, pharyngeal residues, laryngeal penetration and/or laryngotracheal aspiration. OD confirmed by FEES OD assessment: anamnesis, indirect and direct clinical swallowing examination, performance whilst feeding, FEES Medical diagnosis: adults with neurogenic OD (Parkinson’s disease, Ischemic stroke) **Inclusion:** individuals with ischemic stroke, PD undergoing Levodopa treatment **Exclusion:** Individuals on pharmacotherapies, unable to perform the ES manoeuvre, individuals with cardiac arrhythmias, respiratory or endocrine conditions, and impairments that prevent meeting the study requirements; individuals with cognitive impairments that hinder understanding the guidelines for the tasks; and those with more than 5% artifacts during RR interval recording.	N = 22 (patients with neurogenic OD [Parkinson: *n* = 14; stroke: *n* = 8], same participants) categorised to: - **Condition 1** (*n* = 22)**:** ES manoeuvre - **Condition 2** (*n* = 22)**:** Spontaneous swallow manoeuvre	**Demographics** Age *(yrs):* Mean ± SD (Range) Gender *(n_M_/n_F_)*	**Group** 66.24 ± 9.53 (52–89) * PD *6/8*, Stroke *7/1*			
			* Data from supplementary material. Manuscript abstract reports age range 41–75 years.				
**Heslin & Regan, 2022** [70] **Ireland** **III-2** **Strong: 86%**	OD definition: N/R OD confirmed by VFSS OD assessment: FOIS, VFSS Medical diagnosis: adults with OD of mixed aetiology. **Inclusion:** dysphagia (minimum Functional Oral Intake Scale < 6) and a recent VFSS (<1 month). **Exclusion:** pharyngeal or esophageal diverticulum confirmed by VFSS, head and neck cancer, tracheostomy, or recent nasal surgery.	N = 15 (patients with OD of mixed aetiology, same participants) categorised to: - **Condition 1** (*n* = 15)**:** ES manoeuvre - **Condition 2** (*n* = 15)**:** non-ES manoeuvre	**Demographics** Age *(yrs):* Mean (Range) Gender *(n_M_/n_F_)*	**Group** 63.0 (45–86) 8/7			
			Medical diagnoses (N = 15): stroke (*3*), achalasia (*1*), multiple sclerosis (*2*), respiratory failure (*1*), unknown (*2*), autoimmune disease (*1*), chronic obstructive pulmonary disease (*1*), gastroesophageal reflux disease (*1*), lung cancer (*1*), motor neuron disease (*2*)				
**Ko, Shin et al., 2021** [71] **Republic of Korea** **III-2** **Strong: 95%**	OD definition: dysphagia symptoms including drooling and poor oral management, food and/or liquids leaking from the nasal cavity, globus sensation in the neck, complaints of pain when swallowing, wet or gurgly sounding voice during or after eating or drinking, and coughing during or right after eating or drinking OD confirmed by VFSS Medical diagnosis: adults with OD of mixed aetiology. **Inclusion:** penetration into the laryngeal vestibule (VFSS); both neutral and chin tuck positions. **Exclusion:** presence of aspiration (VFSS); inappropriate chin tuck posture.	N = 76 (adults with OD of mixed aetiology, retrospective study, same participants) categorised to: - **Condition 1** (*n* = 76)**:** Chin tuck - **Condition 2** (*n* = 76)**:** Neutral head position	Demographics * Age (*yrs*): Mean ± SD Gender *(n_M_/n_F_)*	**Group (*n* = 26)** **Effective Chin tuck** 65.62 ± 17.66 14/12		**Group (*n* = 50)** **Ineffective Chin tuck** 68.22 ± 12.19 29/21	
			Medical diagnoses (*n* = 76): stroke (*31*), idiopathic (*25*), traumatic brain injury (8), Parkinson’s disease (3), amyotrophic lateral sclerosis (*1*), dermatomyositis (*1*), chronic subdural haemorrhage (*1*), hypoxic brain damage (*1*), epilepsy (*1*), laryngeal cancer (*1*), meningitis (*1*), polymyositis (*1*), tonsillar cancer (*1*) * Total group (N = 76): N/R				
**Koyama et al., 2017** [72] **Japan** **II** **Strong: 100%**	OD definition: N/R OD confirmed by VFSS Medical diagnosis: adults after stroke **Inclusion**: dysphagia, including hypopharyngeal residue found by VFSS; ability to perform the real or sham exercise according to instructions **Exclusion**: level 1 to 4 on the FOIS and/or pulmonary aspiration with 2 mL of barium water in the VFSS; past or present temporomandibular joint disease and/or HNC; past or present progressive disease-causing dysphagia (e.g., PD)	N = 16 (stroke patients, random allocation): - Group 1 * (*n* = 6): Modified Jaw Opening Exercise (MJOE) + visual biofeedback - Group 2 * (*n* = 6): Isometric jaw closing exercise + visual biofeedback (Sham exercise) * Excluding lost to follow-up: *n* = 2 (for each group)	**Demographics** Age *(yrs)*: Mean ± SD Gender *(n_M_/n_F_)* Post onset (*week[s]*) Stroke location: Supra/Infratentorial Lesion: Multiple/Single	**Group 1** 66.0 ± 9.3 5/1 6.7 (2.1) 1/5 2/4		**Group 2** 71.8 ± 7.6 5/1 9.2 (4.0) 1/5 1/5	
**Kumai et al., 2021** [63] **Japan** **III-2** **Strong: 91%**	OD definition: swallowing dysfunction typically characterised by reduced pharyngeal clearance, and silent aspiration (potentially resulting in myasthenic crisis and aspiration pneumonia due to weak oropharyngeal muscle contractions). OD confirmed by FEES Medical diagnosis: adults with MG **Inclusion**: exacerbations of disease stages with difficulty in swallowing, but without aspiration, MG confirmed by neurological evaluation via Myasthenia Gravis Foundation of America (MGFA) clinical classification **Exclusion**: Parkinson’s disease, age > 80 years	N = 7 (MG patients, same participants) categorised to: - **Condition 1** (*n* = 7): Chin-down - **Condition 2** (*n* = 7): Head neutral position	**Demographics** Age *(yrs)*: Mean (Range) Gender *(n_M_/n_F_)* MGFA clinical classification * QMG **: Mean (Range) Oral intake	**Group** 52.6 (35–74) 6/1 IIa (*1*), IIb (*4*), IIIb (*1*), IVa (*1*) 15.0 (10–28) Regular diet 5/7; Soft diet 2/7			
			* Score: I (any muscular weakness) to IV (severe muscular weakness) ** Quantitative Myasthenia Gravis score (total and neck muscles alone)				
**McCullough & Kim, 2012** [40] **USA** **II** **Good: 79%**	OD definition: pharyngeal dysphagia characterised by any apparent reduction in hyolaryngeal elevation or UES opening and evidence of some residue in the pharynx. OD confirmed by VFSS Medical diagnosis: adults after stroke **Inclusion:** over 21 years; post stroke; dysphagic; on restricted diet, defined by need for nasogastric, jejunostomy, or percutaneous endoscopic gastrostomy tube, or an oral diet altered due to swallowing difficulty; minimal functional swallow with some material passing through the UES, aspiration not required. All participants scored 75+ on the Modified Mini-Mental State exam. **Exclusion:** normal swallowing function (determined by VFSS); absent swallow; current or history of tracheotomy; history of swallowing problems before the stroke; progressive neurologic disease; cognitive/physical problems that would impede understanding or completion of therapeutic tasks.	N = 18 (stroke patients, crossover study design, random allocation): - **Condition** 1 (*n* = 9): Mendelsohn manoeuvre with sEMG (2 weeks) - **Condition** 2 (*n* = 9): No treatment (2 weeks)	**Demographics** Age *(yrs)*: Mean (Range) Gender *(n_M_/n_F_)* Post-stroke: Mean (Range)	**Total group (Group 1 + 2)** 70.2 (42–88) 11/7 9.5 months (6 weeks–22 months)			
**McCullough & Kim, 2013** [43] **USA** **II** **Good: 75%**	As per McCullough 2012 (secondary analysis of the same data).	As per McCollough 2012.	As per McCollough 2012.				
**Miyamoto et al., 2021** [76] **Japan** **III-2** **Good: 75%**	OD definition: N/R OD confirmed by VFSS Medical diagnosis: adults with OD of mixed aetiology **Inclusion:** VFSS evaluation at hospital, dysphagia with presence of penetration or aspiration confirmed by VFSS, trial of both neutral and chin tuck completed. **Exclusion:** unable to follow instruction or VFSS procedure due to cognitive impairment or aphasia; no control of head and neck position; unable to sit on a chair during VFSS; incomplete protocols of VFSS.	N = 64 (patients with OD of mixed aetiology, retrospective study, same participants) categorized to: - **Condition 1** (*n* = 64)**:** Chin-down - **Condition 2** (*n* = 64)**:** Head neutral	**Demographics** Age (*yrs*) Gender *(n_M_/n_F_)*	**Group** N/R N/R			
			Medical diagnoses (*n =* 64): head and neck *(26)*, digestive disorder (*20*), neuromuscular disorder (*12*), other (*6*)				
**Nagura et al., 2022** [73] **Japan** **III-2** **Strong: 82%**	OD definition: N/R OD confirmed by VFSS OD assessments: VFSS, Dysphagia Severity Scale (DSS) Medical diagnosis: adults with OD of mixed aetiology **Inclusion**: abnormalities such as aspiration or deep penetration, both with and without head flexion (VFSS). **Exclusion**: inadequate image recording (VFSS).	N = 73 (patients with OD of mixed aetiology, retrospective study, same participants) categorized to: - **Condition 1** (*n* = 73)**:** Head flexion posture - **Condition 2** (*n* = 73)**:** No head flexion posture	**Demographics** Age (*yrs*): Mean ± SD Gender *(n_M_/n_F_)* DSS *: level (*n*)	**Group** 67 ± 14 56/41 2 (*4*), 3 (*39*), 4 (*28*), 5 (*1*), 6 (*1*)			
			Medical diagnoses (*n* = 73): stroke (*33*), cancer (*13*), respiratory disease (*10*), neuromuscular disease (*6*), others (*11*) * Levels 1–4: aspiration (saliva, food, water, occasional), Levels 5–7: without aspiration (oral problems, minimum problems, within normal limits)				
**Ra et al., 2014** [64] **Republic of Korea** **III-2** **Strong: 91%**	OD: N/R OD confirmed by VFSS Medical diagnosis: adults with OD of mixed aetiology (mainly neurogenic) **Inclusion**: dysphagia, presence of penetration or aspiration confirmed by VFSS, trial of chin tuck during VFSS completed. **Exclusion**: unable to follow instructions or difficulty in VFSS procedure due to cognitive impairment or aphasia; no control of head and neck position; unable to sit on a chair during VFSS; incomplete VFSS protocols.	N = 97 (patients with OD of mixed aetiology dysphagia, same participants) categorized to: - **Condition 1** (*n* = 97)**:** Chin tuck - **Condition 2** (*n* = 97)**:** Head neutral position	**Demographics** Age (*yrs*): Mean ± SD Gender *(n_M_/n_F_)*	**Group** **Total group (*n* = 97)** 67.1 ± 13.7 56/41	Group (*n* = 19) Effective Chin tuck (EFF) 64.1 ± 17.7 6/13		Group (*n* = 78) Ineffective Chin tuck (INEFF) 67.8 ± 12.6 50/28
			Medical diagnoses (*n* = 97): stroke (*59*), traumatic brain injury (*10*), Parkinson’s disease (*4*), Guillain-Barre syndrome (2), vocal cord palsy (*2*), hypoxic brain damage (*2*), myasthenia gravis (*1*), hypopharyngeal cancer (*1*), brain metastasis of lung cancer (*1*), bacterial meningitis (*1*), unknown (*14*)				
**Shaker et al., 2002** [75] **USA** **II** **Strong: 89%**	OD definition: pharyngeal phase dysphagia secondary to abnormal upper esophageal sphincter opening OD confirmed by VFFS OD assessment: VFSS, FOAMS (Functional Outcome Assessment of Swallowing) Medical diagnosis: adults with OD of mixed aetiology (mainly stroke and post-pharyngeal radiation). **Inclusion**: abnormal UES opening (post-swallow residue and aspiration), able to independently perform the exercise. **Exclusion**: a history of cervical spine surgery, inability to independently perform the exercise, and patients with tracheostomy.	N = 27 * (random allocation): - **Group 1** (*n* = 27?): Head-raising exercise protocol - **Group 2** (*n* = 7): Passive tongue lateralisation exercise protocol (sham exercise) * Following clinical observation that patients significantly improved in Group 1 (head-raising exercise) but not in Group 2 (sham exercise), no further participants were allocated to the sham group. Additional patients were enrolled in Group 1. Ultimately, all 27 participants completed the head raising exercise protocol, with 7 patients completing the sham exercise prior to participating in the head raising exercise protocol.	**Demographics** Age *(yrs)*: Median (Range) Gender (*n_M_/n_F_*): Duration of dysphagia (*days)*: Mean (Range)	**Total group (N = 27)** 74 (62–89) 25/2 259 (9–2880)			
**Terré & Mearin, 2012** [74] **Spain** **II** **Good: 71%**	OD definition: N/R; Aspiration: entry of food or liquid into the airway below the true vocal folds OD/aspiration confirmed by VFSS OD assessment: VFSS Medical diagnosis:adult neurogenic dysphagia due to stroke or TBI **Inclusion:** VFSS diagnosis of tracheal aspiration (before the onset of pharyngeal swallow and during pharyngeal contraction), acquired brain injury (stroke, TBI), ability to understand and follow verbal instructions for performing the manoeuvre; TBI patients with RLFC score > 412 (Rancho Los Amigos Level Cognitive Function Scale); stroke patients with a Barcelona test (comprehension) score > 15.13 **Exclusion:** cognitive behavioural alteration preventing patients from following instructions; inability to perform the cervical flexion manoeuvre.	N = 72 (patients with neurogenic OD [stroke: *n* = 45; TBI: *n* = 27], crossover study design comparing chin-down vs. no chin-down posture, random allocation): - **Group 1** (*n* = 47): Aspiration group - **Group 2** (*n* = 25): No aspiration group	**Demographics** Age *(yrs)*: Mean (Range) Gender *(n_M_/n_F_)* Stroke/TBI *(n)*	**Group 1** 43 (18–75) 31/16 31/16		**Group 2** 51 (21–76) 19/6 14/11	
**Vose et al., 2019** [65] **USA** **II** **Strong: 82%**	OD definition: presence of a laryngeal vestibule closure (LVC) impairment confirmed by VFSS, PAS > 5, which bolus types led to aspiration or deep laryngeal penetration (safe/unsafe boluses) OD confirmed by VFSS Medical diagnosis: adult OD due to bilateral or unilateral cortical and/or subcortical stroke **Inclusion:** any history of stroke (confirmed by CT or MRI scan during medical chart review), age 21–100 years, deep penetration or aspiration on at least one bolus type. **Exclusion:** dementia (MMSE score < 22), cerebellar infarct, oral apraxia, pregnancy, chronic obstructive pulmonary disease (COPD) or serious respiratory illness.	N = 19? * (volitional laryngeal vestibule closure [vLVC] manoeuvre using three different biofeedback modalities, random allocation): - **Group 1** (*n* = 6)**: ssEMG** (submental surface EMG) - Groups 2 * (*n* = 6)**: VF** - Groups 3 * (*n* = 6)**: Mixed (ssEMG + VF)** * Inconsistencies reporting sample sizes. Original sample size N = 21. Lost to follow-up (*n* = 3) due to failure to demonstrate vLVC manoeuvre (Group 2: *n* = 2; Group 3 *n* = 1).	**Demographics** Age *(yrs)*: Mean ± SD Gender (*n_M_/n_F_*) Type of stroke *(n)*	**Total group (N = 19?)** 58 ± 18 13/6 ischemic (*7*); hemorrhagic (*7*); embolic (*4*); TIA (*1*)			

^a^ NHMRC hierarchy [55] Level I Systematic reviews; Level II Randomised control trials; Level III–1 Pseudo-randomised control trials; Level III–3 Comparative studies with historical control, two or more single-arm studies, or interrupted time series without a control group; Level IV Case series. ^b^ Methodological quality (QualSyst) [56]: strong > 80%; good 60–79%; adequate 50–59%; poor < 50%. ^c^ Group 1: single behavioural intervention group, Group 2: study or comparison group. Notes. b/w—between; CT—Computer Tomography; CTAR—chin tuck against resistance; CVA—cerebrovascular accident; ES—Effortful swallow; FEES—Fiberoptic Endoscopic Evaluation of Swallowing; FOIS—Functional Oral Intake Scale; HNC—Head and Neck Cancer; M/F—male/female; IQR—Interquartile range; MG—myasthenia gravis; mL—milliliter; MMSE—Mini-Mental Status Examination; MRI—Magnetic Resonance Imaging; N/R—not reported; PAS—Penetration-Aspiration Scale; PD—Parkinson disease; RAT—radiotherapy; R/L—right/left; sEMG—surface electromyography; SD—standard deviation; SLT—speech and language therapist; TBI—Traumatic Brain Injury; UES—upper esophageal sphincter; VF—video fluoroscopy; VFSS—videofluoroscopic swallowing study; yrs—years; ?—Uncertain or unclear study data.

**Table 3:** Outcome of behavioural interventions for people with oropharyngeal dysphagia.

Author, Year	Intervention Goals	Intervention Delivery and Dosage ^a^	Materials and Procedures ^a^	Outcome Measures	Treatment Outcomes ^a^ (Main Outcome According to Authors)
**Archer et al., 2021** [39]	To determine: (1) if age or stroke-related dysphagia affects the ability to increase submental muscle activity during ES compared to normal swallowing; (2) if sEMG biofeedback improves the performance of ES by healthy and dysphagic stroke participants; (3) if participants find sEMG comfortable, helpful and acceptable.	**Intervention agent** sEMG biofeedback and swallow task training provided by researchers. **Delivery/Dosage** 2 sessions completed within 1 week, but >24 h apart. Each session consisted of 3 NS and 6 ES tasks repeated twice, with a 5 min rest. Two conditions: with and without biofeedback. Swallows were facilitated by 5 mL water bolus, with 30 s rests b/w each.	**Summary:** ES (with or without biofeedback) vs. normal swallowing (NS) in stroke vs. healthy participants. **All participants** followed the same procedure: they were instructed to perform ES with the command “Swallow hard, squeezing all of your throat muscles and pushing hard with your tongue on the roof of your mouth”. For the NS participants were told to “Swallow in your normal way”. Each participant received a 5 mL water bolus from a teaspoon and was instructed to hold water in their mouth until prompted to swallow. If there was a high risk of aspiration, accommodations (e.g., consistency modification or moistened mouthcare swabs) were made, with the same bolus type used across sessions. Participants were randomly exposed to either: “with biofeedback followed by without biofeedback” or “without biofeedback followed by with biofeedback”. **EMG biofeedback condition**: participants saw the Digital Swallow Workstation (DSW) screen while performing swallowing tasks and were verbally encouraged to increase the strength of each ES using visual targets to “beat” (cursors placed on their previous attempt). No additional instruction was given for NS. **Non-biofeedback condition**: participants did not see the DSW screen and were verbally encouraged to swallow “harder” during ES.	**Primary outcomes** *Submental muscle activity* - sEMG amplitudes. **Secondary outcomes** *Comfort, utility and acceptability* of sEMG biofeedback—questionnaire with 8 questions about participant impression of completing the ES with and without sEMG biofeedback.	**Primary outcomes** - Both groups produced significantly greater muscle activity for the ES than NS (*p* < 0.001) and significantly increased activity with biofeedback (*p* < 0.001) with no effect of age. - Healthy controls produced greater muscle activity than stroke participants in all conditions and sessions (*p* < 0.001). **Secondary outcomes** - 86% of stroke participants and all healthy controls found sEMG helpful in completing the tasks. ES is a physiologically beneficial dysphagia exercise that enhances muscle activity during swallowing, with sEMG biofeedback further improving performance and being well-tolerated by patients.
**Don Kim et al., 2015** [66]	To examine the effects of short neck flection exercises using PNF on the swallowing function of stroke patients with dysphagia.	**Intervention agent** 2 experienced PTs (Physical therapists) **Delivery/Dosage** Group 1 (isometric, isotonic Shaker exercises): 3 days a week for 30 min. each time over a period of 6 weeks. Group 2 (PNF-based short neck flexion exercises): 3 days a week for 30 min each time over a period of 6 weeks.	**Summary:** PNF-based short neck flexion exercise vs. (isometric/isotonic) Shaker exercise in stroke patients. **Group 1**: **(a) Isometric Shaker exercise:** patients lay on a bed, raised their heads without moving their shoulders, and looked at the ends of their feet for 60 s, then lowered their heads back on the bed and rested for 60 s. If a patient struggled, they performed the exercise for as long as possible, repeating it 3x. **(b) Isotonic Shaker exercise:** patients raised their heads in the same posture and looked at the ends of their feet for 30 consecutive repetitions. If unable to complete 30, fewer repetitions were allowed. **Group 2:** PNF-based short neck flection exercises: Patients lie on a bed with their heads and necks off the edge. A tester, positioned on the left side behind the patient’s head, supports the patient’s left laryngeal region with his right hand and places his left fingertips below the patient’s jaw. Patients are instructed to look at a target object 15° diagonally to the right. The tester moves the patient’s neck diagonally in the opposite direction, instructing them to: “draw your jaw inward” while applying resistance to the jaw to activate the neck flexors (performing external cervical flexion). The patient performs cervical flexion or right-side rotation. If the patient struggles, the tester provides light support instead of resistance to help complete the exercise. The exercises are repeated in the opposite direction.	**Primary outcomes** *VFSS* - New VFSS scale - ASHA NOMS scale (American Speech-Language-Hearing Association National Outcomes Measurement System scale)	**Primary outcomes** - Significant improvements in Group 1: premature bolus loss, residue in the valleculae, laryngeal elevation, epiglottic closure, residue in the pyriform sinuses, pharyngeal transit time, and aspiration on both the new VFSS scale and the ASHA NOMS scale (*p* < 0.05). - Significant improvements in Group 2: premature bolus loss, residue in the valleculae, laryngeal elevation, epiglottic closure, residue in the pyriform sinuses, coating of the pharyngeal wall after swallowing, pharyngeal transit time, and aspiration on both the new VFSS scale and the ASHA NOMS scale (*p* < 0.05). - Statistically N/S differences b/w the groups in the ASHA NOMS scale and the new VFSS scale results.
**Forbes & Humbert, 2021** [67]	To examine the impact of chin-down on swallowing physiology in patients with various medical conditions and swallowing impairments. To determine: (1) if swallowing kinematics are affected by chin-down; (2) if chin-down improves airway protection and bolus efficiency.	**Intervention agent** practising clinician **Delivery/Dosage** 1–2 VFSS sessions	**Summary:** Chin down posture vs. neutral head position in OD patients of mixed aetiology (same participants). **VFSS examination:** Neutral head position (no chin down) or chin-down (if physiologically necessary in aberrant bolus flow). Swallowed bolus: 5 mL of room-temperature thin liquid barium, at least one swallow in both positions. The number of swallows: 1–5 per patient. Areas visualised: oral cavity, velum, pharynx, hyoid bone, larynx, UES, cervical esophagus, and cervical vertebrae.	**Primary outcomes** *VFSS* - Bolus depth zones at time of hyoid burst - Swallowing kinematics: STD (stage transition duration); dLVC (duration of laryngeal vestibule closure); dtLVC (B)—(duration to laryngeal vestibule closure [bolus]); dtLVC (H)—(duration to laryngeal vestibule closure [hyoid]); dtUESO (B)—(duration to upper esophageal sphincter opening [bolus]); dtUESO (H)—(duration to upper esophageal sphincter opening [hyoid]); PTT (pharyngeal transit time); dUESO (duration of upper esophageal sphincter opening) - PAS	**Primary outcomes** - Increased time b/w swallow onset and entry of a prematurely spilled bolus into the pharynx (STD-early release) in condition 1 (chin-down) versus condition 2 (neutral head position) (*p* = 0.006). - N/S difference in bolus depth zone location, bolus efficiency kinematics (PTT, dtUESO, dUESO) or airway protection (STD and PAS) between conditions. Chin-down posture inconsistently prevented the presence of penetration and aspiration within and across patients.
**Fraser & Steele, 2012** [68]	To study the impact of the chin down on swallowing safety (penetration and aspiration) in a general acute care patient population (stroke and general internal medicine).	**Intervention agent** SLP **Delivery /Dosage** Single session VFSS	**Summary:** Chin down position vs. head neutral position with two bolus administrations (teaspoon/cup-sip) in 2 groups of patients (stroke, general internal medicine -GIM). **VFSS examination:** 1. In head neutral position (no chin down); a teaspoon of thin liquid barium was swallowed; 2. If penetration/aspiration occurred, chin down (by tucking the head downward, “looking down to the knees”) and the same bolus volume was given; 3. If no issues are found, proceed to the cup-drinking task with a thin liquid barium in a neutral head position (with the chin up); 4. If penetration/aspiration occurs, the chin is down, and the same bolus is administered from a cup. VFSS completed. VFSS recordings captured with the image field from the lips to the upper esophagus.	**Primary outcomes** *VFSS* - Chin angle (extent of head flexion) - PAS: 1 (normal), 2 (high penetration), 3–5 (penetration) and 6–8 (aspiration)	**Primary outcomes** - N/S group differences (stroke vs. GIM) for frequency of airway invasion in any of the four conditions. (Aetiology factor removed from subsequent analyses.) - *Chin-down position (administration)* - Statistically significant differences for airway invasion between administration methods, with a greater tendency towards worse airway invasion scores in the teaspoon-administration condition (*p =* 0.000). The chin-down position improved airway protection in patients with impaired swallowing safety during cup drinking of thin liquids but not with teaspoon-sized bolus administration. - *Head* neutral vs. Chin *down position* (*n*: Normal, High penetration, Penetration, Aspiration) Teaspoon Neutral: 0, 68, 26, 5. Teaspoon Chin Down: 0, 32, 42, 26. Cup drinking Neutral: 0, 47, 30, 23. Cup drinking Chin Down: 37, 50, 10, 3.
**Gomes et al., 2020** [69]	To evaluate the effects of ES on cardiac autonomic control (heart rate variability—HRV) in subjects with neurogenic OD.	**Intervention agent** N/R **Delivery/Dosage** Spontaneous swallow: 5 min at rest, 3 spontaneous swallows of saliva every 1 min and 30 s. ES: 3 ESs, each one in 1 min and 30 s during 5 min.	**Summary:** ES vs. spontaneous swallow in neurogenic OD patients (same participants). **ES training protocol**: Involved 3 ES (tongue force on the palate), each lasting 1 min and 30 s, performed during 5 min. **Experimental procedures:** Split into two randomised stages, via card allotments on the same day: **(a) Spontaneous swallowing:** Subjects rested for 5 min, then performed 3 spontaneous swallows of saliva on command every 1 min 30 s, with additional swallowing as needed. **(b) ES**: After the initial 5 min, subjects performed the training ES for an additional 5 min, completing 3 ES every 1 min 30 s, as directed. HRV: Compared between spontaneous swallowing and the ES protocol.	**Primary outcomes** *HRV analysis* - HR: heart rate - RMSSD: square root of the square mean of differences b/w adjacent normal inter-beat intervals (RR) in a time interval - HF: high-frequency band - SD1: standard deviation of instantaneous beat-to-beat variability **Secondary Outcome** *sEM*G (Surface electromyographic) evaluation	**Primary outcomes** - HR: no changes in HRV b/w two spontaneous swallowing events (8.68 ± 13.91 bpm vs. ES 102.57 ± 107.81 bpm, *p* = 0.201). - RMSSD: spontaneous swallowing 16.99 ± 15.65 ms vs. ES 44.74 ± 138.85 ms, *p* = 0.312. - HF: spontaneous swallowing 119.35 ± 273 ms2 vs. ES 99.83 ± 194.58 ms2, *p* = 0.301. - SD1: spontaneous swallowing 12.02 ± 1.07 ms vs. ES 31.66 ± 98.25 ms, *p* = 0.301. - ES did not cause clinically significant changes in the autonomic control of HR in subjects with neurogenic OD. Swallowing parameters (HRV analysis) showed no clinically significant changes in autonomic heart rate control during ES in subjects with OD.
**Heslin & Regan, 2022** [70]	To quantify the effects of ES on pharyngeal swallowing biomechanics in adults with OD using pharyngeal high-resolution manometry (PHRM).	**Intervention agent** Clinical SLP **Delivery/Dosage** Single session HRM	**Summary:** ES vs. non-ES swallow in mixed aetiology OD patients (same participants, randomised order of condition). **ES procedure:** 2 × 10 mL liquid boluses administered via 20 mL syringe and swallowed under two conditions: non-ES (control) and ES. Verbal cues prior to each trial: “swallow as normal” and “squeeze hard with all your muscles as you swallow” (ES). Where post-swallow piecemeal deglutition and coughing occurred, a trial was repeated. Minimum 30 s period between swallows was enforced to prevent inhibition of esophageal peristalsis.	**Primary outcomes** *High-resolution manometry (HRM)* - Pressure and timing metrics (velopharyngeal, mesopharyngeal, hypopharyngeal and upper esophageal sphincter [UES] regions)	**Primary outcomes** Significantly increased metrics during ES vs. non-ES: - Global pharyngeal pressure (*p* = 0.002). - Velopharyngeal pressure (*p* = 0.001). - Mesopharyngeal pressure (*p* = 0.027). - Hypopharyngeal contractile integral (HPCI) (*p* = 0.003), observed in 87% participants during ES. - UES relaxation time (*p* = 0.002). - N/S difference in UES integrated relaxation pressure. ES significantly increased global pharyngeal contractility during swallowing thin liquids in adults with OD, supporting that ES generates higher pharyngeal pressure than non-ES.
**Ko, Shin et al., 2021** [71]	To investigate, via VFSS, the effect of the chin tuck on the severity of penetration.	**Intervention agent** Physiatrist experienced in VFSS analysis **Delivery/Dosage** Single session VFSS	**Summary:** Comparison of chin tuck vs. head neutral position in mixed aetiology OD patients (same participants, retrospective study). **VFSS examination**: 3 mL boluses in the following order: 3 × thick liquid (International Dysphagia Diet Standardisation Initiative—IDDSI 3); 2 × Rice porridge (IDDSI 2); 2 × curd yogurt (IDDSI 1); 3 × thin liquid (IDDSI 0); 2 × 5 mL of thin liquid from a cup. If penetration occurred in the head neutral position, the patient performed the chin tuck manoeuvre (“tuck chin as close to sternum as possible”), with the same consistency and volume. Repeat if the chin tuck posture was incorrect. **VFSS analysis:** Selected frames showing deepest penetration, with contrast visible between the laryngeal inlet and vocal fold. Measurements conducted using ImageJ^®®^ software. The Chin tuck manoeuvre is considered effective if the penetration severity is reduced by at least one grade.	**Primary outcomes** *VFSS* - Penetration ratio: ratio penetration depth to epiglottis length (five levels: 1 very shallow—5 very deep) - Oral phase parameter: completeness of lip closure; presence of oral residue; presence of premature bolus leakage, OTT (oral transit time). - Pharyngeal phase parameters: delayed triggering of pharyngeal swallowing; height of laryngeal elevation; residues in the valleculae and pyriformis sinuses; PDT (pharyngeal delayed time), PTT.	**Primary outcomes** - Statistically significant decrease in the penetration depth (*p* = 0.000) and ratio (*p* = 0.001) in chin tuck vs. neutral position. - Decreased residues in both effective and ineffective groups when using chin tuck, with residues in the vallecular and pyriformis sinuses less severe in the effective group. - N/S differences for the other parameters between conditions, and between the effective and ineffective groups.
**Koyama et al., 2017** [72]	To verify the feasibility of MJOE and its effectiveness in promoting anterior displacement of the hyoid bone during swallowing in stroke patients with OD.	**Intervention agent:** MJOE trainer (not specified). **Delivery/Dosage** Group 1 (MJOE): maintain 80% maximum voluntary contraction (MVC) for 6 s, 5x repetitions (1 set), 4 sets/day, 5x/week, 6 weeks total, 6 weeks resistive load Group 2 (Sham exercise): 20% MVC for 6 s, 5x repetitions (1 set), 4 sets/day, 5 x/week, 6 weeks total, 6 weeks resistive load	**Summary:** MJOE vs. Sham exercise in stroke patients. **Group 1 (MJOE):** Surface electrodes on SHMs at the mandibular midline, connected to biofeedback equipment. Participants were asked to close their mouths in a comfortable sitting position, with the front half of their tongues pressed against the hard palate. The trainer placed a hand under the participant’s chin and applied upward vertical resistance. Visual feedback was provided on the intensity of the isometric opening movement of the mandible (mouth closed, front half of tongue pressed against the hard palate). Participants were instructed to maintain 80% MVC during exercise. **Group 2 (Sham exercise: Isometric jaw closing exercise)**: Surface electrodes attached to the masseter muscle and connected to biofeedback equipment. Participants were given visual feedback on the intensity of the isometric closing movement of the mandible (jaw occluded in a comfortable sitting position); then instructed to maintain 20% MVC during exercise. Discontinuance criteria: pain in the TMJ (Temporomandibular Joint) and/or anterior region of the neck during exercise.	**Primary outcomes** *VFSS* - DMH: distance b/w the mental spine and the hyoid bone **Secondary outcomes** *VFSS* - HD: hyoid displacement - Pharyngeal Residue	**Primary outcomes** - DMH (end position of anterior displacement) intragroup—significant decrease seen in Group 1 (*p* = 0.046), N/S difference in Group 2; intergroup—significant decrease in Group 1 (*p* = 0.041). **Secondary outcomes** - HD (anterior) intragroup—increased significantly in Group 1 (*p* = 0.028), N/S difference in Group 2; intergroup—significant increase in Group 2 (*p* = 0.002). - Pharyngeal residue: significant improvement in piriform sinus in Group 1 (*p* = 0.001), N/S difference seen in Group 2. MJOE is feasible without any adverse events in poststroke patients, and it promotes anterior HD during swallowing.
**Kumai, Miyamoto et al., 2021** [63]	To compare oropharyngeal swallowing dysfunction in MG patients with difficulty in swallowing between the neutral and chin-down positions using high-resolution manometry (HRM).	**Intervention agent** N/R **Delivery/Dosage** Single session HRM	**Summary:** Chin-down vs. head neutral positions in MG patients (same participants). **HRM examination:** Patients examined at the same time period (4–6 PM). HRM utilized to identify each portion in typical pressure topography data. Representative data from MG patients with mild to moderate OD were assessed in both conditions: head neutral position and chin-down position. Averaged data over 3 swallows: maximum SP at velopharynx, meso–hypopharynx, and UES, and duration of UES relaxation pressure.	**Primary outcomes** HRM parameters: - Maximum swallowing pressure (SP) at the soft palate, meso-, hypopharynx, and UES - Duration of relaxation SP at UES. **Secondary outcomes** - QMG (Quantitative Myasthenia Gravis), grip strength, head elevation movement test	**Primary outcomes** - Statistically significant changes between chin down vs. neutral position: increased maximum SP at the meso–hypopharynx (*p* = 0.07), decreased resting and maximum SP at the UES (*p* = 0.039), and increased duration of UES relaxing SP (*p* < 0.016). **Secondary outcomes** - N/S correlations between the SP at any location and neurological evaluations (secondary outcomes). The chin-down position appears useful for improving pharyngeal clearance in MG patients, by promoting increased SP at the meso–hypopharynx, relaxing SP at the UES, and increasing the duration of UES relaxation.
**McCullough et al., 2012** [40]	To determine any lasting changes in swallowing physiology following intensive exercise using the Mendelsohn Manoeuvre in stroke patients with dysphagia.	**Intervention agent:** Principal investigator with some support from the study clinician. **Delivery/Dosage:** Twice daily sessions, 45–60 min, with a 2–3 h break b/w sessions, 2 weeks total.	**Summary:** Mendelsohn manoeuvre vs. no treatment in stroke patients (crossover study design) **Treatment sessions**: Participants were taught the Mendelsohn manoeuvre (squeezing and holding the larynx at the peak of the swallow) using surface electromyography (sEMG) feedback. Non-adjustable, ground, and active electrode pads placed submentally at the midline, halfway b/w the mental symphysis and the hyoid bone tip. Tracings were used to provide participant biofeedback. Before each swallow, dental swabs dipped in ice water were used to moisten the mouth, facilitating swallowing. Clinician feedback—video information provided to participants included onset/offset points, swallow duration, peak amplitude (from Prometheus software) and comparisons of duration to the previous swallow. Aimed for 40 swallows but stopped after a minimum of 30 if discomfort occurred.	**Primary outcomes** *VFSS* - DOHME (duration of hyoid maximum elevation); DOHMAE (duration of hyoid maximum anterior excursion); DOUESO (duration of UES opening) **Secondary outcomes** *VFSS* - OTD (oral transit duration); PTD (pharyngeal transit duration); TSD (total swallow duration); STD 1 STD 2; PRD (pharyngeal response duration); DTOUES (duration to open UES) - PAS - Oropharyngeal residue scale - Dysphagia Outcome and Severity Scale (DOSS)	**Primary outcomes** - All duration measures improved during treatment weeks and deteriorated during no-treatment weeks. DOHME & DOHMAE improvements were significant, respectively, *p* = 0.011 and *p* = 0.009 at 2 weeks post-treatment. No other results were significant. **Secondary Outcomes** - PRD improved during treatment periods/deteriorated during no-treatment periods (*p* value N/R); difference N/S. No other measures found improvements during treatment weeks. Intensive use of the Mendelsohn manoeuvre altered the duration of hyoid movement and UES opening.
**McCullough & Kim, 2013** [43]	To determine the effects of the Mendelsohn manoeuvre on the distance the hyoid travels superiorly and anteriorly, and the impact on the mean width of the UES opening in stroke patients with dysphagia.	Secondary data analysis of McCullough 2012.	Secondary data analysis of McCullough 2012.	**Primary outcomes** *VFSS* - Hyoid movement: MHE (maximum hyoid elevation), MHAE (maximum hyoid anterior excursion), MWUESO (mean width of UES opening) - Hyoid duration/Bolus flow: DOHME; DOHMAE; DOUESO - **Secondary outcomes** *Dose response*	**Primary outcomes** - All distance/displacement measures increased more after treatment weeks than after no-treatment, differences were N/S (*p* = 0.135). - Increased consistency significantly affected MWUESO (increased UES opening O width) *p* = 0.03. - Univariate follow-up testing highlighted the relative strength of MHE compared to changes in other measures *F*(1.139) = 4.337, *p* = 0.02 **Secondary outcomes** - N/S differences b/w no-treatment and treatment weeks (MHAE, *p* = 0.33; MHE, *p* = 0.07; MWUESO, *p* = 0.08). All measures increased slightly after 1 week of treatment and then decreased after 2 weeks of no treatment. After Mendelsohn manoeuvre training, gains were demonstrated in the extent of hyoid movement and UES opening, as well as improvements in the coordination of structural movements with each other and with bolus flow.
**Miyamoto, Kumai et al., 2021** [76]	To evaluate pharyngeal swallowing to determine the characteristics of OD (pathophysiology and type of disease) that respond positively to the chin-down and help prevent aspiration.	**Intervention agent:** 3 expert SLPs **Delivery/Dosage:** Single session VFSS	**Summary:** Comparison of chin-down vs. head-neutral position in mixed aetiology OD patients (same participants, randomised order of condition). **VFSS examination:** 3 × 3 mL bolus of barium sulphate 120 *w*/*v*% (<30 s time intervals between swallows) in both positions: head neutral position and chin-down (“neck flexion” position, the neck flexed at the level of the lower cervical spine). For high-risk aspiration patients (PAS 7–8), swallowing was performed once.	**Primary outcomes** *VFSS* - Modified Barium Swallow Impairment Profile (MBSImP): Presence and Degree of Airway Invasion (PDAI), tongue base retraction, anterior hyoid movement, soft palate elevation, laryngeal elevation and closure, UES opening, pharyngeal swallowing initiation, bolus residue in the pyriform sinus and vallecula	**Primary outcomes** - Statistically significant improvement (*p* < 0.01–0.05) chin down vs. neutral position for the following parameters: PDAI, pharyngeal constriction, anterior hyoid movement, laryngeal elevation, laryngeal closure, UES opening, initiation of the pharyngeal swallow and pharyngeal clearance - Diagnostic subgroup analyses for chin down vs. neutral position: statistically significant improvement (*p* < 0.01) on PDAI in patients with head and neck (laryngeal elevation and closure) and digestive (laryngeal closure). Insufficient laryngeal closure due to inadequate laryngeal elevation is the pathophysiology most likely to respond favourably to the chin-down manoeuvre.
**Nagura, Kagaya et al., 2022** [73]	To assess the effects of head flexion posture in patients with dysphagia of mixed aetiology.	**Intervention agent:** Research technician experienced in image analysis **Delivery/Dosage:** Single session VFSS	**Summary:** Head flexion posture vs. without head flexion posture in mixed aetiology OD patients (same participants, retrospective study). **VFSS examination:** Head flexion posture (“Bring the chin as close as possible to the neck without flexing the neck”); no head flexion posture. Evaluation of biomechanical aspects of swallowing, including timing of swallow and head and neck flexion angles. Thickness and amount of contrast material were changed according to the patient’s condition (same type and amount of bolus for both postures); bolus consistency: thin liquid (*n* = 45) and thick liquid (*n* = 28); bolus volume: 4 mL (*n* = 44) and 10 mL (*n* = 19); Position: sitting (*n* = 66) and at 75°, 60°, and 45° angles from the supine position (*n* = 1, 5 and 1, respectively).	**Primary outcomes** *VFSS* - Head and neck flexion angles (at the initiation of the swallowing reflex) - PAS (including presence or absence of aspiration) - Nasopharyngeal closure time; STD; laryngeal closure duration; time from initiation of swallow reflex to initiation of laryngeal closure and UES opening; UES opening duration; position of the leading edge of bolus at initiation of swallowing reflex; bolus transition time	**Primary outcomes** Statistically significant changes for head flexion compared to no head flexion: - Increased head flexion angle (*p* < 0.001), but N/S changes neck flexion angle. - Improved PAS score and reduced aspiration (*p* < 0.001). - Reduced laryngeal closure duration (*p* = 0.006): time from initiation of swallow reflex to initiation of laryngeal closure. - Shallower leading bolus edge at initiation of swallowing reflex (*p* = 0.004). N/S changes for remaining outcome variables. Head flexion posture resulted in earlier laryngeal closure and a shallower position of the leading bolus edge at the swallowing reflex, leading to PAS improvement and decreased aspiration.
**Ra et al., 2014** [64]	To identify factors affecting the efficiency of the chin tuck and determine the optimal neck flexion angle in chin tuck in patients with OD of mixed aetiology.	**Intervention agent:** Physiatrist experienced in VFSS analysis **Delivery/Dosage:** Single VFSS session.	**Summary:** Comparison of chin tuck vs. head neutral position in mixed aetiology OD patients (same population). **VFSS examination:** Head neutral position: 5 × 5 mL thin bolus barium. Chin tuck pose (flexion of the head as much as possible, touching the chin to the chest) in case of penetration/aspiration: repeat 5 × 5 mL same bolus.	**Primary outcomes** *VFSS* - Oral phase parameters: lip closure completeness, oral residue, premature bolus leakage, OTT - Pharyngeal parameters: delay of triggering pharyngeal swallowing, height of laryngeal elevation, presence of the residue in the valleculae and pyriform sinuses, PDT, PTT. - 8PPAS (8-point penetration-aspiration scale) - Neck flexion angle (optimal neck flexion angle in decreasing penetration and aspiration)	**Primary outcomes** Chin tuck vs. head neutral: - Decrease (improvement) of more than one scale in 8PPAS in 19.6% (19/97). - Significantly shortened OTT, PDT and PTT. Efficient (EFF) vs. inefficient (INEFF) chin tuck: - Significant mean group differences in 8PPAS scores: −3.3 ± 2.7 vs. 0.9 ± 1.8 (*p* < 0.001). - N/S group differences for chin angle at neutral and chin tuck, and lordosis angle at chin tuck position. Chin tuck might be less effective in those who have excessive residue in the pyriformis sinus. Sufficient neck flexion is important: the required minimum neck flexion is 17.5° for benefitting from the chin tuck.
**Shaker et al., 2002** [75]	To evaluate the effect of the head-raising exercise on the deglutitive biomechanical events and functional outcome of swallowing in patients with deglutitive failure due to abnormal UES opening, necessitating PEG (Percutaneous Endoscopic Gastrostomy).	**Intervention agent:** SLP **Delivery/Dosage:** 3 x/day × 6 weeks	**Summary:** Head-raising vs. Sham exercise in pharyngeal dysphagia. **Group 1 (Head-raising exercise protocol)**: Lie flat and perform 3 sustained head raisings for 1 min in the supine position, with a 1 min rest period. Followed by 30 consecutive repetitions of head raising in the same position (raise head enough to observe toes without raising shoulders). **Group 2 (Sham protocol)**: 15 repetitions of passive tongue lateralisation.	**Primary outcomes** *VFSS* - Anterior–posterior/lateral UES opening diameter; maximum superior/anterior hyoid excursion; maximum superior/anterior laryngeal excursion; pyriform sinus residue (height/width); aspiration *FOAMS* (Functional Outcome Assessment of Swallowing)	**Primary outcomes** - Group 2: No change in swallow function was observed following 6 weeks of sham exercise. - Group 1: All patients demonstrated significant improvement in UES opening, anterior laryngeal excursion after 6 weeks of exercise (*p* < 0.01) and experienced resolution of post-deglutitive aspiration. Similar results were observed in the 7 patients who crossed over from Group 2 to Group 1. A suprahyoid muscle strengthening exercise program is effective in restoring oral feeding in some patients with deglutitive failure because of abnormal UES opening.
**Terré & Mearin, 2012** [74]	To assess the effectiveness of the chin-down posture to prevent aspiration in neurogenic dysphagia patients secondary to acquired brain injury (stroke and trauma).	**Intervention agent:** Researcher **Delivery/Dosage:** Single VFSS session.	**Summary:** Chin-down vs. no chin-down in neurogenic dysphagia patients, with or without aspiration (crossover study). **VFSS examination:** no chin-down (cervical rachis in the anatomic position); chin-down (cervical rachis in flexion with sterno-mental contact). Evaluated biomechanical swallow with 3, 5, 10, and 15 mL boluses of pudding, nectar, and liquid consistencies in both positions. Swallowing sequences were recorded; oral and pharyngeal function was assessed for all viscosities and volumes, including cricopharyngeal dysfunction and pharyngeal residue where applicable. Examination discontinued if the patient aspirated or was unable to cooperate.	**Primary outcomes** *VFSS* - Aspiration - Oral phase parameters: OTT; tongue control; reduced palatoglossal closure; piecemeal deglutition - Pharyngeal parameters: nasopharyngeal penetration; pharyngeal residue (vallecula and pyriform sinuses); laryngeal elevation; cricopharyngeal dysfunction; PDT; PTT; penetration/aspiration. - Sensitivity and specificity of the chin-down manoeuvre to prevent tracheal aspiration. (Se = probability of patients swallowing in the chin-down posture compensating the aspiration; Sp = probability of patients swallowing with cervical rachis in the anatomic position—without flexion—not compensating the aspiration)	**Primary outcomes** - Group 1 (aspiration group): 55% of patients avoided aspiration during the chin-down manoeuvre. When aspiration occurred before swallowing, it was prevented by the chin-down posture in 40% of cases, whereas when aspiration occurred during pharyngeal contraction, it was prevented in 60%. Silent aspiration was identified in 51% of patients (20% before and 80% during swallowing), with the chin-down posture preventing aspiration in 52% of these cases. - Several VFFS parameters were associated with manoeuvre inefficiency, highlighting the need to assess the indication for the chin-down posture through VFSS. - Sensitivity and specificity: 65% and 54%, respectively.
**Vose et al., 2019** [65]	To compare the effect of 3 visual biofeedback conditions (ssEMG; VF, mixed VF + ssEMG) on swallowing airway protection accuracy when training the vLVC (volitional laryngeal vestibule closure) swallowing manoeuvre in poststroke patients with dysphagia, and to examine clinician accuracy in judging vLVC performance.	**Intervention agent:** Experienced clinicians. **Delivery/Dosage:** Maximum of 20 saliva and 40 bolus swallows. Saliva swallows: 3 s in duration for 3 swallows, increased by 1 s up to a maximum of 6 s (where possible, dependent on patient tolerance and fatigue). Rest periods approx. 10 s. Bolus swallows followed same pattern.	**Summary:** vLVC using three types of biofeedback (ssEMG, VF and mixed) in stroke patients. **Phase 1—Accurate demonstration of vLVC** **Manoeuvre:** clinician provided instructions and demonstration of vLVC manoeuvre. Participants performed the exercise to feel superior movement during a saliva swallow, focusing on differences in hyolaryngeal elevation duration. Next, they were instructed to swallow and hold their thyroid notch up as high and as long as possible during swallowing, and to ensure that they could not breathe during the manoeuvre, given closure of the vestibule. **Phase 2—vLVC Manoeuvre training:** A series of saliva vLVC swallows followed by bolus vLVC swallows, using ssEMG, VF, or combination (Mixed). Participants viewed the vLVC in a normal saliva swallow in real-time. They were then instructed to perform the vLVC manoeuvre while watching real-time ssEMG/VF. The clinician provided verbal cues in real time. - **Group 1 (ssEMG**): ssEMG signals were displayed on a screen. Participants were shown that increased amplitude indicated more muscle activity and instructed to sustain that amplitude for at least 2 s. - **Group 2 (VF)**: VF images were displayed on a monitor, with attention drawn to relevant anatomy and structural airway changes during normal swallowing, emphasizing laryngeal vestibule kinematics. - **Group 3 (Mixed: ssEMG + VF):** (VF—phase 1, ssEMG—phase 2): Participants received the same orientation to VFSS and ssEMG as Group 1 and 2. - **Group 1** (*n* = 6)**: ssEMG** (submental surface EMG) - **Groups 2** (*n* = 6)**: VF** - **Groups 3** (*n* = 6)**: Mixed (ssEMG + VF)**	**Primary outcomes** *VFSS* - Accuracy of vLVC movements during training. - Accuracy of prolonging vLVC for the target duration during training (dichotomous scale) - Accuracy of clinical verbal cues (comparison of clinician judgment and VFSS analysis; dichotomous scale)	**Primary outcomes** - Group 1 showed significantly worse accuracy in vLVC training performance and clinician feedback, as well as in prolonging vLVC for the target duration, compared to Groups 2 and 3 (*p* < 0.001). Accuracy rates: 2% for Group 1, 46% for Group 2, and 43% for Group 3. - A significant difference in clinician accuracy was observed b/w Group 1 and Group 2 (*p* < 0.001), as well as b/w Group 1 and Group 3 (*p* < 0.001). Accuracy rates: 40% for Group 1, 80% for Group 2, and 74% for Group 3. Both the accuracy of vLVC training performance and clinician feedback were poorer in the ssEMG group (1) compared to the VF group (2) and mixed groups (3).

^a^ Group 1: single behavioural intervention group, Group 2: study or comparison group. Note. ASHA—The American Speech-Language-Hearing Association; ASHA NOMS—(The American Speech-Language-Hearing Association) National Outcomes Measurement System; b/w—between; cP: centipoise; DOHMAE—duration of hyoid maximum anterior excursion; DOHME—duration of hyoid maximum elevation; DOUESO—duration of UES opening; EMG—electromyography; ES—effortful swallow; FEES—Fibreoptic Endoscopic Evaluation of Swallowing; HRM—high-resolution manometry; min—minute/s; MG—myasthenia gravis; MJOE—Modified Jaw Opening Exercise; mL—milliliter; N/S—not significant; NS—normal swallowing; OTT—oral transit time; PAS—Penetration-Aspiration Scale; PDT—pharyngeal delay/delayed time; PNF—Proprioceptive neuromuscular facilitation; PTT—pharyngeal transit time; sec—second/s; sEMG—Surface electromyography; SLP—speech language pathologist; sEMG –surface electromyography; ssEMG—submental surface electromyography; STD—stage transition duration; UES—upper esophageal sphincter; VF—videofluoroscopy, videofluoroscopic; VFSS—videofluoroscopic swallowing study; vLVC—volitional laryngeal vestibule closure.

### 3.3. Methodological Quality

According to the standard quality assessment tool (QualSyst) [56], six of the included studies [40,43,66,69,74,76] were classified as having good methodological quality with overall quality percentage scores ranging from 71% to 79%. The remaining 11 studies [39,63,64,65,67,68,70,71,72,73,75] were classified as having strong methodological quality, with scores ranging from 82% to 100%.

In accordance with the NHMRC guidelines [55], this systematic review included eight level II studies or randomised controlled trials [39,40,43,65,66,72,74,75]; one level III-1 study or pseudo-randomised controlled trial [68]; and eight Level III-2 studies classified as comparative studies with concurrent controls [63,64,67,69,70,71,73,76].

### 3.4. Effect of Interventions

*Main Results* (Table 3): Of the three studies examining the effects of the *effortful swallow manoeuvre*, two [39,70] demonstrated a significant increase in muscle activity during the manoeuvre compared to normal swallowing, with sEMG biofeedback further enhancing performance in one study [39]. A third study [69] corroborated these findings regarding increased muscle activity; however, it did not identify any association with clinically meaningful changes in the autonomic regulation of heart rate. Due to heterogeneity in outcome measures and insufficient reporting of data, no meta-analysis was performed. Two related studies reported on the *Mendelsohn manoeuvre.* One utilised primary data analysis [40], and the other secondary data analysis of the same dataset [43]. In both studies, measures of hyoid duration improved during treatment weeks and declined during no-treatment weeks. The results showed that intensive use of the Mendelsohn manoeuvre changed the duration of hyoid movement and had a positive effect on upper esophageal sphincter opening.

Similarly, the study assessing the *modified jaw-opening exercise* MJOE [72] demonstrated improved anterior hyoid displacement during swallowing compared to a control group performing sham exercises. One study evaluating the impact of different types of biofeedback on the performance of the *volitional laryngeal vestibule closure* (vLVC) manoeuvre [65] reported greater patient performance accuracy and clinician feedback accuracy when using videofluoroscopic visual feedback alone or combined with ssEMG, compared to the use of ssEMG alone, thereby demonstrating the value of kinematic or videofluoroscopic biofeedback. Additionally, two studies [66,75] investigated the *Shaker or Head-raising exercises*, both demonstrating significant improvements in oral feeding and swallowing function. However, no improvements were observed after sham training [75], whereas both proprioceptive neuromuscular facilitation (PNF)-based short neck flexion exercises and isometric/isotonic Shaker exercises yielded positive results [66]. A meta-analysis was not conducted due to variations in study designs. However, the eight studies on the chin tuck provided sufficient data for a meta-analysis, which is outlined below.

*Meta-Analysis*: Of the eight studies reporting on the chin tuck [63,64,67,68,71,73,74,76], three were excluded from the meta-analysis. One study [64] provided insufficient data for quantitative synthesis; another [76] included only patients with evidence of penetration or aspiration, whereas the other studies enrolled populations at risk of these events. A third study was excluded to reduce heterogeneity, as it was the only study using high-resolution manometry (HRM), while all others relied on visuoperceptual evaluation of VFSS recordings. One study [68] that reported on the same participants under different conditions (chin tuck during teaspoon administration vs. cup drinking), the effects were combined for inclusion in the forest plot.

Risk of bias was evaluated using the Begg and Mazumdar rank correlation test and the Fail-safe N. The Begg and Mazumdar test produced a tau of 0.300 (two-tailed *p* = 0.462), indicating no evidence of publication bias. The meta-analysis, involving five studies, yielded a *z*-value of 7.414 (two-tailed *p* < 0.001). The Fail-safe N was 67, meaning that 67 additional ‘null’ studies would be needed for the combined two-tailed *p*-value to surpass 0.050. In other words, 13.4 unpublished studies per included study would be required to nullify the observed effect. Overall, these results from both the Begg and Mazumdar test and the Fail-safe N suggest that publication bias is unlikely.

**Figure 2:**
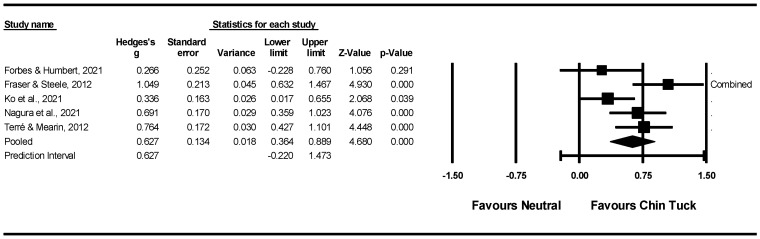
Within intervention group pre-post meta-analysis [67,68,71,73,77].

Overall, the pooled within-group analysis using a random-effects model revealed a significant, moderate intervention effect for the chin tuck (*z*(4) = 4.680, *p* < 0.001, Hedges’ *g* = 0.672, and 95% CI = 0.364–0.889), with a prediction interval of −0.220 and 1.473. Although all effects indicated improvement compared to the normal swallow or head-neutral position (Figure 2), effect sizes varied between 0.266 and 1.049 across the studies. Of the five intervention groups included in the meta-analysis, one group showed large effect sizes (Hedges’ *g* > 0.8), two groups showed moderate effect sizes (0.5 < Hedges’ *g* ≤ 0.8), and two groups showed small effect sizes (0.2 < Hedges’ *g* ≤ 0.5).

## 4. Discussion

### 4.1. Swallowing Manoeuvres, Exercises and Postural Strategies

The objective of this systematic review was to assess the efficacy of the most frequently employed swallowing manoeuvres, exercises, and postural strategies as standalone behavioural interventions for the management of OD in adults, without limiting the population based on underlying medical diagnoses. In contrast to prior reviews, this study explicitly focused on the effect of individual intervention components when utilised independently, facilitating a more precise evaluation of each component’s specific impact.

Despite comprehensive literature searches, only seventeen studies met the inclusion criteria, encompassing a range of distinct interventions, including the chin tuck [63,64,67,68,71,73,74,76], effortful swallow [39,69,70], Mendelsohn manoeuvre [40,43], Modified Jaw Opening Exercise (MJOE) [72], volitional laryngeal vestibule closure (vLVC) [65], and Shaker or Head-raising exercises [66,75]. Most studies reported favourable treatment outcomes, thereby endorsing the advantageous effects of both compensatory techniques (e.g., chin tuck) and diverse rehabilitative interventions across various populations, with the majority of evidence drawn from mixed populations (nine studies) or individuals who have experienced a stroke (six studies). However, one study [67] confirmed inconsistent prevention of penetration and aspiration both within and across patients when using the chin down posture, whereas another [68] reported significant positive effects of the manoeuvre during cup drinking, but no significant changes when administered by teaspoon. Overall, considering the limited number of studies and their substantial heterogeneity, a meta-analysis was conducted solely for the chin tuck, indicating that the promising evidence remains preliminary. This focus reflects data availability rather than comparative efficacy. Future research should generate robust, standardised evidence across compensatory techniques to reduce heterogeneity and enable balanced evaluation of their effectiveness.

During the PRISMA selection process, numerous studies were excluded due to low-quality study designs and the absence of a control group. However, populations could potentially serve as their own control group using crossover study designs or by comparing normal swallowing to the application of a compensatory intervention within the same subjects. Studies employing these methodologies were also deemed eligible for inclusion. Additionally, many studies investigated combined therapies (i.e., therapies involving multiple postural strategies and/or swallowing manoeuvres or exercises), where the outcomes of individual strategies or manoeuvres could not be isolated from the overall effects of the therapy and, as a result, were excluded from this review.

Although it is common practice for clinicians to employ a variety of intervention strategies when treating patients with diverse swallowing problems and underlying diagnoses, these conventional or ‘black box’ interventions hinder the ability to identify the effects of individual treatment components. The only way to disentangle the effects of individual behavioural components, as well as potential interaction effects between these components, involves conducting large-scale, high-quality studies that account for heterogeneity in patient characteristics, intervention protocols, and outcome measures. Such studies are presently absent. A recent review [13] evaluating randomised clinical trials reporting on behavioural interventions in people with OD categorised conventional dysphagia treatment into compensatory, rehabilitative, and combined compensatory and rehabilitative interventions. However, no distinction was made between individual components or the effects of standalone behavioural interventions.

The seventeen studies incorporated within this review validated the existence of OD among participants through instrumental assessment, in accordance with the specified inclusion criteria. Nonetheless, there was limited information regarding the operational definition of dysphagia. The majority of studies did not provide additional details [64,67,68,70,72,73,76], whereas some offered only broad descriptions, such as difficulty with swallowing and/or mastication [39,66].

One study presented a comprehensive symptom list [63], whereas the remaining studies referred to specific videofluoroscopic characteristics or biomechanical events [40,43,65,69,71,74,75]. A more widely accepted definition of OD, such as the one proposed by Speyer et al. [2] based on an internationally established Delphi consensus, would improve comparability across research. Additionally, the included studies primarily relied on instrumental assessments, such as VFSS, and occasionally used sEMG or HRM to evaluate the effects of therapy. Nevertheless, given that OD is considered a multidimensional phenomenon, both non-instrumental clinical assessments conducted by dysphagia experts and patient-reported measures (e.g., health-related quality of life and functional health status) should also be considered when evaluating therapy outcomes [78]. The wide variety of assessment methods across studies contributes to heterogeneity, emphasising the need for standardised outcome measures in research. To improve comparability and further reduce heterogeneity, future studies should aim for more consistent use of standardised outcome measures for selected outcomes, with outcomes referring to the aspects of health or function being evaluated (e.g., swallowing safety or quality of life) and outcome measures referring to the tools or instruments used to assess each specific outcome.

### 4.2. Limitations

This study employed a consistent review process, including systematic database searches, screening of records and reports by multiple independent reviewers following PRISMA guidelines [53], and a thorough methodological quality assessment (QualSyst, NHMRC) [55,56]. Nevertheless, certain limitations persist. Some studies may have been inadvertently omitted or overlooked during the review process. Furthermore, the restriction of inclusion criteria to publications in English may have led to the exclusion of pertinent non-English studies. Additionally, this review concentrated on the most prevalent postural strategies and swallowing manoeuvres or exercises, which may have resulted in the exclusion of relevant but less common behavioural interventions.

Variability in study design, small sample sizes, heterogeneous patient populations, differing durations of interventions, and a wide array of outcome measures diminished the comparability and generalisability of the findings. Notably, numerous reports were excluded due to the presence of intervention combinations, which impacted the final count of included studies. Due to the limited number of standalone interventions and significant heterogeneity among the studies, a meta-analysis was conducted solely for the chin tuck, suggesting that the current evidence remains preliminary.

## 5. Conclusions

This systematic review sought to evaluate the effectiveness of the most commonly used swallowing manoeuvres, exercises, and postural strategies when used as standalone interventions in the behavioural treatment of OD in adults. Consequently, combined therapies, which involve multiple postural strategies and/or swallowing manoeuvres or exercises, were excluded as the outcomes of individual strategies or manoeuvres could not be distinguished from the overall therapeutic effects. Although few studies satisfied the eligibility criteria, the majority reported positive treatment outcomes, indicating the potential benefits of both compensatory and rehabilitative interventions. Nevertheless, owing to the limited number of studies and significant heterogeneity among them, a meta-analysis was performed solely for the chin tuck manoeuvre, resulting in a moderate positive intervention effect. Therefore, the current evidence remains preliminary and should be interpreted with caution.

There is a need for future research to focus on standalone behavioural interventions for adult OD, emphasising the importance of high-quality study designs and larger sample sizes to disentangle the effects of individual behavioural components and potential interaction effects between them. This research should also account for heterogeneity in patient characteristics, intervention protocols, and outcome measures. The utilisation of more advanced statistical methodologies will facilitate more robust conclusions regarding the efficacy of these interventions.

## Figures and Tables

**Table 1:** Search strategies per database.

Search Strategies
**Embase:** (swallowing/ OR dysphagia/) AND (chin tuck OR chin-tuck OR chin down OR chin-down OR head flexion OR neck flexion OR head tilt forward OR head tilt OR flexion of the head OR head rotation OR head-rotation OR head turn OR rotation of the head OR rotated head OR head back OR chin up OR head extension OR extension of the head OR head tilt OR tilting head OR side-lying OR side lying OR Mendelsohn OR supraglottic swallow OR voluntary airway closure OR airway closure technique OR airway protection technique OR airway protection maneuver OR airway protection manoeuvre OR breath-holding OR breath holding OR supersupraglottic swallow OR super-supraglottic swallow OR super supraglottic swallow OR effortful swallow OR hard swallow OR Masako OR Tongue holding OR tongue-holding OR tongue hold OR tongue-hold OR Shaker OR head lift)
**PubMed:** (“Deglutition”[Mesh] OR “Deglutition Disorders”[Mesh]) AND (“chin tuck” OR “chin-tuck” OR “chin down” OR “chin-down” OR “head flexion” OR “neck flexion” OR “head tilt forward” OR “head tilt” OR “flexion of the head” OR “head rotation” OR “head-rotation” OR “head turn” OR “rotation of the head” OR “rotated head” OR “head back” OR “chin up” OR “head extension” OR “extension of the head” OR “head tilt” OR “tilting head” OR “side-lying” OR “side lying” OR “Mendelsohn” OR “supraglottic swallow” OR “voluntary airway closure” OR “airway closure technique” OR “airway protection technique” OR “airway protection maneuver” OR “airway protection manoeuvre” OR “breath-holding” OR “breath holding” OR “supersupraglottic swallow” OR “super-supraglottic swallow” OR “super supraglottic swallow” OR “effortful swallow” OR “hard swallow” OR “Tongue holding” OR “tongue-holding” OR “Masako” OR “tongue hold” OR “tongue-hold” OR “Shaker” OR “head lift”)

## Data Availability

No new data were created or analyzed in this study.

## Supplementary Materials

The following supporting information can be downloaded at: https://www.mdpi.com/article/10.3390/jcm14207180/s1, Supplementary Table S1: Common swallowing manoeuvres, exercises, and postural strategies. Supplementary File S1: PRISMA 2020 Abstract checklist. Supplementary File S2: PRISMA 2020 Checklist. Supplementary File S3: PRISMA-S Checklist.
