# Supplementary material for: Evaluating Behavioural Interventions for Oropharyngeal Dysphagia in Adults: A Systematic Review and Meta-Analysis of Swallowing Manoeuvres, Exercises, and Postural Techniques
Source: J Clin Med. 2025 Oct 11;14(20):7180. doi: 10.3390/jcm14207180 (PMC12564565; doi:10.3390/jcm14207180)
Supplement: Supplementary file 1 [file jcm-14-07180-s001.zip › Supplementary Table S1 Common swallowing manoeuvres, exercises and postural strategies.pdf]

## Supplementary Table S1. Common swallow manoeuvres, exercises and postural strategies.

| Treatment approach | Synonyms (Similar terms) | Deficits | Purpose | Description |
|---|---|---|---|---|
| **Swallow manoeuvre** | | | | |
| **Effortful swallow** (compensatory and rehabilitative) | Effortful swallow<br>Hard swallow<br>(manoeuvre / technique) | - decreased tongue base retraction<br>- decreased oropharyngeal pressure | To improve pharyngeal bolus propulsion, reducing residue in the valleculae and/or pyriform sinuses and aspiration [1-3]:<br>- by strengthening the muscles involved in swallowing (tongue, infrahyoid, suprahyoid),<br>- by increasing tongue base retraction and posterior pharyngeal wall movement during the swallowing,<br>- by increasing orolingual and pharyngeal bolus pressure generation [1,4]. | Voluntary swallow with maximal effort (with or without bolus):<br>- achieved by forcefully squeezing the tongue and throat muscles [4] or by pushing the tongue hard against the hard palate [1] |
| **Mendelsohn manoeuvre** (compensatory and rehabilitative) | Mendelsohn | - decreased movement of the hyolaryngeal complex<br>- discoordination of swallowing[5,6] | To enhance bolus propulsion through the pharynx, reducing pharyngeal residue and aspiration [7-9]:<br>- by volitional increase in the duration of submental muscle contraction,<br>- by improving the coordination of the swallowing muscles [10,11]. | Squeezing the swallow at the apex [7,8].<br>Begin swallowing normally. When the larynx reaches its highest point, voluntarily hold that position for a few seconds before relaxing and completing the swallow [12] |
| **Supraglottic swallow** (compensatory and/or rehabilitative) | Voluntary airway closure<br>Breath-holding manoeuvre / technique / strategy | Decreased airway protection at the level of glottis [13,14] | To increase airway protection and reduce the risk of aspiration during the swallow [15]:<br>- by closing the airway at the level of the vocal folds before swallowing [5,8,13,16,17]. | A voluntary breath-hold technique performed without extra effort:<br>Inhale and hold the breath before and during the swallow. Cough at the end of the swallow (before inhaling) [12,18]. |
| **Super supraglottic swallow** (compensatory and/or rehabilitative) | As per Supraglottic swallow | Decreased airway protection before and during swallowing at the level of supraglottis [6] | To increase airway protection and reduce the risk of aspiration before and during the swallow [19]:<br>- by closing the airway at the level of the supraglottis (laryngeal vestibulum and vestibular folds) before and during swallowing [6,20,21]. | A voluntary breath-hold technique performed with the forceful effort.<br>Inhale, bear down and hold the breath before and during the swallow. Cough at the end of the swallow (before inhaling) [19,22]. |
| **Swallow exercise** | | | | |
| **Chin tuck against resistance (CTAR)** (rehabilitative) | Modified Shaker exercise | - reduced movement of the hyolaryngeal complex<br>- abnormal UES opening [23] | To improve swallowing ability, bilateral vallecular and pyriform sinus residue clearance and reduce post-swallow aspiration:<br>- by strengthening the suprahyoid muscles using resistance,<br>- by promoting hyo-laryngeal complex elevation,<br>- by reducing strain on the neck muscles [23-25]. | In a sitting position the chin is tucked downward against a resistance tool (e.g. rubber ball, neckline slimmer device):<br>- Isometric CTAR: hold a sustained chin tuck against resistance<br>- Isokinetic CTAR: perform a series of consecutive chin tucks against resistance [23-25]. |
| **Jaw opening against resistance (JOAR)** (rehabilitative) | Modified Shaker exercise | - reduced movement of the hyolaryngeal complex<br>- abnormal UES opening [24] | To improve pharyngeal passage, thus pharyngeal residue [26]:<br>- by strengthening and increasing the volume of the suprahyoid muscles,<br>- by strengthening the jaw-opening muscles,<br>- by increasing upward movement of the hyoid bone,<br>- by improving both the extent of UES opening and the duration of pharynx passage [24-26]. | Isometric JOAR<br>in a sitting position with holding a sustained jaw opening against resistance:<br>the jaw is opened against resistance, pressing mandible downward as forcefully as possible for 10 seconds [27,28]. |

| | | | | |
|---|---|---|---|---|
| **Masako manoeuvre** (rehabilitative) | Tongue holding / Tongue hold swallow manoeuvre / exercise | - decreased tongue base retraction<br>- decreased pharyngeal constriction [3] | To increase pharyngeal driving pressure, enhance bolus propulsion, and improve pharyngeal transit speed, thereby reducing residue [29]:<br>- by increasing tongue base retraction and promoting contact between the tongue base and the anterior bulging of the posterior pharyngeal wall,<br>- by training pharyngeal constrictors during swallowing [3,29-31]. | Tongue hold.<br>Slightly protrude the tongue between the teeth (or gums), gently bite down to secure it in place, and swallow saliva while maintaining this position [29,30,32] |
| **Shaker exercise** (rehabilitative) | Head lift exercise<br>Head raise exercise | - reduced movement of the hyolaryngeal complex<br>- abnormal UES opening [33] | To improve swallowing ability, bilateral pyriform sinus residue clearance and reduce post-swallow aspiration:<br>- by strengthening the suprahyoid muscles and promoting hyo-laryngeal complex elevation,<br>- by increasing UES opening [33-35]. | Lie in a supine position and lift the head to look at toes.<br>- Isometric head lift: maintain a sustained static head lift.<br>- Isokinetic head lift: perform a series of consecutive head lifts [36,37]. |
| **Postural strategy** | | | | |
| **Chin tuck** (compensatory) | Chin down posture<br>Chin down movement<br>Chin to chest<br>Chin tuck exercise<br>(Forward) flexion of the head<br>Head / Neck / Head and neck flexion<br>Head tilt forward | Delayed:<br>- oral control,<br>- swallowing trigger,<br>- tongue base retraction [38]<br>Deficit in the vocal fold closure [39] | To improve airway protection, reduce laryngeal penetration and aspiration before and during swallowing [40,41]:<br>- by keeping the bolus in the oral cavity longer, facilitated by gravity,<br>- by narrowing the laryngeal entrance, improving tongue-base and posterior pharyngeal wall contact, and reducing laryngo-hyoid and hyoid-mandibular distances [16,17,42-45],<br>by facilitating the opening of the upper esophageal sphincter (UES) [46]. | The chin is lowered prior to swallowing (“look down”) |
| **Chin up** (compensatory) | Head / Neck extension<br>Extension of the head<br>Head back (posture) | - oral & lingual deficits<br>oral transfer deficits [39,47] | To facilitate the pharyngeal phase of swallowing [46]:<br>- by improving bolus propulsion using gravity,<br>- by mechanically widening the laryngeal entrance and narrowing the valleculae,<br>by decreasing the duration of the UES relaxation [48,49]. | The chin is elevated prior to swallowing (“look up”) |
| **Head tilt** (compensatory) | Head tilt (posture)<br>Head tilt to stronger / healthy / intact side<br>Tilting head | - Unilateral oral and/or pharyngeal dysfunction, deficits [5,50,51] | To facilitate poor unilateral oral clearance, reduce unilateral post-swallow pharyngeal residue and aspiration risk [22,52,53]:<br>- by directing bolus toward the side of the head tilt (along the healthy side or the side with better function), while closing the weaker side of the pharynx<br>by increasing lower pharyngeal pressure [52]. | The head is tilted toward the healthy or stronger side prior to swallowing<br>(“ear to the shoulder”) |
| **Head turn** (compensatory) | Head rotation towards the weak / damaged / affected side<br>Head-rotation posture<br>Head turn (manoeuvre) | - Unilateral pharyngeal deficits [54,55] | To facilitate swallowing, improve bolus flow control, reduce unilateral post-swallow residue and aspiration risk [51]:<br>- by rotating the head toward the affected side, reducing the anatomical space of the pyriform sinus and redirecting the bolus to the opposite, stronger, more sensitive side of the pharynx [50,52],<br>by prolonging the UES opening and lowering its pressure on the opposite side of head rotation [52]. | The head is rotated toward the lateral damaged or weak side prior to swallowing<br>(“look over the shoulder”) |
| **Side lying** (compensatory) | Side-lying on the stronger side<br>Side-lying posture | Unilateral hypopharyngeal dysfunction or deficit<br>- (dysmotility, contractibility deficit) [5] | To improve bolus flow control and minimize unilateral pharyngeal residue and the post-swallow aspiration risk:<br>- by altering gravity to direct residue toward the stronger, more sensitive pharyngeal wall and pyriform sinus, facilitating its clearance with additional swallows [56-58]<br>- by increasing hypopharyngeal pressure and maximum opening of the pharyngesophageal sphincter | Lying on the stronger, more sensitive side prior to swallowing |

**References:**


1. Fukuoka, T.; Ono, T.; Hori, K.; Tamine, K.-i.; Nozaki, S.; Shimada, K.; Yamamoto, N.; Fukuda, Y.; Domen, K. Effect of the effortful swallow and the Mendelsohn maneuver on tongue pressure production against the hard palate. *Dysphagia* **2013**, *28*, 539-547.
2. Hind, J.A.; Nicosia, M.A.; Roecker, E.B.; Carnes, M.L.; Robbins, J. Comparison of effortful and noneffortful swallows in healthy middle-aged and older adults. *Arch Phys Med Rehabil* **2001**, *82*, 1661-1665, doi:10.1053/apmr.2001.28006.
3. Lazarus, C.; Logemann, J.A.; Song, C.W.; Rademaker, A.W.; Kahrilas, P.J. Effects of voluntary maneuvers on tongue base function for swallowing. *Folia Phoniatr Logop* **2002**, *54*, 171-176, doi:10.1159/000063192.
4. Jang, H.J.; Leigh, J.H.; Seo, H.G.; Han, T.R.; Oh, B.M. Effortful swallow enhances vertical hyolaryngeal movement and prolongs duration after maximal excursion. *J Oral Rehabil* **2015**, *42*, 765-773, doi:10.1111/joor.12312.
5. Speyer, R. *Behavioural treatment of oropharyngeal dysphagia: bolus modification and management, sensory and motor behavioural techniques, postural adjustments, and swallow manoeuvres.*; Medical Radiology: diagnostic imaging. Springer: Heidelberg, Germany, 2012.
6. van der Kruis, J.G.; Baijens, L.W.; Speyer, R.; Zwijnenberg, I. Biomechanical analysis of hyoid bone displacement in videofluoroscopy: a systematic review of intervention effects. *Dysphagia* **2011**, *26*, 171-182, doi:10.1007/s00455-010-9318-9.
7. Kahrilas, P.J.; Logemann, J.A.; Krugler, C.; Flanagan, E. Volitional augmentation of upper esophageal sphincter opening during swallowing. *Am J Physiol* **1991**, *260*, G450-456, doi:10.1152/ajpgi.1991.260.3.G450.
8. Lazarus, C.; Logemann, J.A.; Gibbons, P. Effects of maneuvers on swallowing function in a dysphagic oral cancer patient. *Head Neck* **1993**, *15*, 419-424, doi:10.1002/hed.2880150509.
9. Wheeler-Hegland, K.; Ashford, J.; Frymark, T.; McCabe, D.; Mullen, R.; Musson, N.; Hammond, C.S.; Schooling, T. Evidence-based systematic review: Oropharyngeal dysphagia behavioral treatments. Part II--impact of dysphagia treatment on normal swallow function. *J Rehabil Res Dev* **2009**, *46*, 185-194.
10. Inamoto, Y.; Saitoh, E.; Ito, Y.; Kagaya, H.; Aoyagi, Y.; Shibata, S.; Ota, K.; Fujii, N.; Palmer, J.B. The Mendelsohn Maneuver and its Effects on Swallowing: Kinematic Analysis in Three Dimensions Using Dynamic Area Detector CT. *Dysphagia* **2018**, *33*, 419-430, doi:10.1007/s00455-017-9870-7.
11. McCullough, G.H.; Kamarunas, E.; Mann, G.C.; Schmidley, J.W.; Robbins, J.A.; Crary, M.A. Effects of Mendelsohn maneuver on measures of swallowing duration post stroke. *Top Stroke Rehabil* **2012**, *19*, 234-243, doi:10.1310/tsr1903-234.
12. Logemann, J.A.; Pauloski, B.R.; Rademaker, A.W.; Colangelo, L.A. Speech and swallowing rehabilitation for head and neck cancer patients. *Oncology (Williston Park)* **1997**, *11*, 651-656, 659; discussion 659, 663-654.
13. Bodén, K.; Hallgren, A.; Witt Hedström, H. Effects of three different swallow maneuvers analyzed by videomanometry. *Acta Radiol* **2006**, *47*, 628-633, doi:10.1080/02841850600774043.
14. Mendelsohn, M.S.; Martin, R.E. Airway protection during breath-holding. *Ann Otol Rhinol Laryngol* **1993**, *102*, 941-944, doi:10.1177/000348949310201206.

15. Kasahara, T.; Hanayama, K.; Kodama, M.; Aono, K.; Masakado, Y. The efficacy of supraglottic swallow as an indirect swallowing exercise by analysis of hyoid bone movement. *Tokai J Exp Clin Med* **2009**, *34*, 72-75.
16. Bülow, M.; Olsson, R.; Ekberg, O. Videomanometric analysis of supraglottic swallow, effortful swallow, and chin tuck in healthy volunteers. *Dysphagia* **1999**, *14*, 67-72, doi:10.1007/pl00009589.
17. Hori, K.; Tamine, K.; Barbezat, C.; Maeda, Y.; Yamori, M.; Müller, F.; Ono, T. Influence of chin-down posture on tongue pressure during dry swallow and bolus swallows in healthy subjects. *Dysphagia* **2011**, *26*, 238-245, doi:10.1007/s00455-010-9292-2.
18. Guedes, R.; Azola, A.; Macrae, P.; Sunday, K.; Mejia, V.; Vose, A.; Humbert, I.A. Examination of swallowing maneuver training and transfer of practiced behaviors to laryngeal vestibule kinematics in functional swallowing of healthy adults. *Physiol Behav* **2017**, *174*, 155-161, doi:10.1016/j.physbeh.2017.03.018.
19. Logemann, J.A.; Pauloski, B.R.; Rademaker, A.W.; Colangelo, L.A. Super-supraglottic swallow in irradiated head and neck cancer patients. *Head Neck* **1997**, *19*, 535-540, doi:10.1002/(sici)1097-0347(199709)19:6<535::aid-hed11>3.0.co;2-4.
20. Martin, B.J.; Logemann, J.A.; Shaker, R.; Dodds, W.J. Coordination between respiration and swallowing: respiratory phase relationships and temporal integration. *J Appl Physiol (1985)* **1994**, *76*, 714-723, doi:10.1152/jappl.1994.76.2.714.
21. Ohmae, Y.; Logemann, J.A.; Kaiser, P.; Hanson, D.G.; Kahrilas, P.J. Effects of two breath-holding maneuvers on oropharyngeal swallow. *Ann Otol Rhinol Laryngol* **1996**, *105*, 123-131, doi:10.1177/000348949610500207.
22. Vose, A.; Nonnenmacher, J.; Singer, M.L.; González-Fernández, M. Dysphagia Management in Acute and Sub-acute Stroke. *Curr Phys Med Rehabil Rep* **2014**, *2*, 197-206, doi:10.1007/s40141-014-0061-2.
23. Park, J.S.; Hwang, N.K. Chin tuck against resistance exercise for dysphagia rehabilitation: A systematic review. *J Oral Rehabil* **2021**, *48*, 968-977, doi:10.1111/joor.13181.
24. Kraaijenga, S.A.; van der Molen, L.; Stuiver, M.M.; Teertstra, H.J.; Hilgers, F.J.; van den Brekel, M.W. Effects of Strengthening Exercises on Swallowing Musculature and Function in Senior Healthy Subjects: a Prospective Effectiveness and Feasibility Study. *Dysphagia* **2015**, *30*, 392-403, doi:10.1007/s00455-015-9611-8.
25. Yoon, W.L.; Khoo, J.K.; Rickard Liow, S.J. Chin tuck against resistance (CTAR): new method for enhancing suprahyoid muscle activity using a Shaker-type exercise. *Dysphagia* **2014**, *29*, 243-248, doi:10.1007/s00455-013-9502-9.
26. Wada, S.; Tohara, H.; Iida, T.; Inoue, M.; Sato, M.; Ueda, K. Jaw-opening exercise for insufficient opening of upper esophageal sphincter. *Arch Phys Med Rehabil* **2012**, *93*, 1995-1999, doi:10.1016/j.apmr.2012.04.025.
27. Choi, J.B.; Jung, Y.J.; Park, J.S. Comparison of 2 types of therapeutic exercise: jaw opening exercise and head lift exercise for dysphagic stroke: A pilot study. *Medicine (Baltimore)* **2020**, *99*, e22136, doi:10.1097/md.0000000000022136.
28. Koyama, Y.; Sugimoto, A.; Hamano, T.; Kasahara, T.; Toyokura, M.; Masakado, Y. Proposal for a Modified Jaw Opening Exercise for Dysphagia: A Randomized, Controlled Trial. *Tokai J Exp Clin Med* **2017**, *42*, 71-78.
29. Fujiu, M.; Logemann, J.A. Effect of a tongue-holding maneuver on posterior pharyngeal wall movement during deglutition. *American Journal of Speech-Language Pathology* **1996**, *5*, 23-30.

30. Doeltgen, S.H.; Witte, U.; Gumbley, F.; Huckabee, M.-L. Evaluation of manometric measures during tongue-hold swallows. *American Journal of Speech-Language Pathology* **2009**.
31. Umeki, H.; Takasaki, K.; Enatsu, K.; Tanaka, F.; Kumagami, H.; Takahashi, H. Effects of a tongue-holding maneuver during swallowing evaluated by high-resolution manometry. *Otolaryngology–Head and Neck Surgery* **2009**, *141*, 119-122, doi:10.1016/j.otohns.2009.01.025.
32. Fujiu-Kurachi, M.; Fujiwara, S.; Tamine, K.-i.; Kondo, J.; Minagi, Y.; Maeda, Y.; Hori, K.; Ono, T. Tongue pressure generation during tongue-hold swallows in young healthy adults measured with different tongue positions. *Dysphagia* **2014**, *29*, 17-24.
33. Easterling, C.; Grande, B.; Kern, M.; Sears, K.; Shaker, R. Attaining and maintaining isometric and isokinetic goals of the Shaker exercise. *Dysphagia* **2005**, *20*, 133-138, doi:10.1007/s00455-005-0004-2.
34. Antunes, E.B.; Lunet, N. Effects of the head lift exercise on the swallow function: a systematic review. *Gerodontology* **2012**, *29*, 247-257, doi:10.1111/j.1741-2358.2012.00638.x.
35. Mepani, R.; Antonik, S.; Massey, B.; Kern, M.; Logemann, J.; Pauloski, B.; Rademaker, A.; Easterling, C.; Shaker, R. Augmentation of deglutitive thyrohyoid muscle shortening by the Shaker Exercise. *Dysphagia* **2009**, *24*, 26-31, doi:10.1007/s00455-008-9167-y.
36. Shaker, R.; Easterling, C.; Kern, M.; Nitschke, T.; Massey, B.; Daniels, S.; Grande, B.; Kazandjian, M.; Dikeman, K. Rehabilitation of swallowing by exercise in tube-fed patients with pharyngeal dysphagia secondary to abnormal UES opening. *Gastroenterology* **2002**, *122*, 1314-1321, doi:10.1053/gast.2002.32999.
37. Shaker, R.; Kern, M.; Bardan, E.; Taylor, A.; Stewart, E.T.; Hoffmann, R.G.; Arndorfer, R.C.; Hofmann, C.; Bonnevier, J. Augmentation of deglutitive upper esophageal sphincter opening in the elderly by exercise. *Am J Physiol* **1997**, *272*, G1518-1522, doi:10.1152/ajpgi.1997.272.6.G1518.
38. Robbins, J.; Butler, S.G.; Daniels, S.K.; Diez Gross, R.; Langmore, S.; Lazarus, C.L.; Martin-Harris, B.; McCabe, D.; Musson, N.; Rosenbek, J. Swallowing and dysphagia rehabilitation: translating principles of neural plasticity into clinically oriented evidence. *J Speech Lang Hear Res* **2008**, *51*, S276-300, doi:10.1044/1092-4388(2008/021).
39. Macrae, P.; Anderson, C.; Humbert, I. Mechanisms of airway protection during chin-down swallowing. *J Speech Lang Hear Res* **2014**, *57*, 1251-1258, doi:10.1044/2014_jslhr-s-13-0188.
40. Matsubara, K.; Kumai, Y.; Miyamoto, T.; Samejima, Y.; Yoshida, N.; Baba, H.; Orita, Y. The effect of a chin-down maneuver after esophagectomy on oropharyngeal swallowing pressure measured using high-resolution manometry. *Auris Nasus Larynx* **2020**, *47*, 141-147, doi:https://doi.org/10.1016/j.anl.2019.06.001.
41. Robbins, J.; Gensler, G.; Hind, J.; Logemann, J.A.; Lindblad, A.S.; Brandt, D.; Baum, H.; Lilienfeld, D.; Kosek, S.; Lundy, D.; et al. Comparison of 2 interventions for liquid aspiration on pneumonia incidence: a randomized trial. *Ann Intern Med* **2008**, *148*, 509-518, doi:10.7326/0003-4819-148-7-200804010-00007.
42. Ashford, J.; McCabe, D.; Wheeler-Hegland, K.; Frymark, T.; Mullen, R.; Musson, N.; Schooling, T.; Hammond, C.S. Evidence-based systematic review: Oropharyngeal dysphagia behavioral treatments. Part III--impact of dysphagia treatments on populations with neurological disorders. *J Rehabil Res Dev* **2009**, *46*, 195-204.

43. Bülow, M.; Olsson, R.; Ekberg, O. Videomanometric analysis of supraglottic swallow, effortful swallow, and chin tuck in patients with pharyngeal dysfunction. *Dysphagia* **2001**, *16*, 190-195.
44. McCabe, D.; Ashford, J.; Wheeler-Hegland, K.; Frymark, T.; Mullen, R.; Musson, N.; Hammond, C.S.; Schooling, T. Evidence-based systematic review: Oropharyngeal dysphagia behavioral treatments. Part IV--impact of dysphagia treatment on individuals' postcancer treatments. *J Rehabil Res Dev* **2009**, *46*, 205-214.
45. Terré, R.; Mearin, F. Effectiveness of chin-down posture to prevent tracheal aspiration in dysphagia secondary to acquired brain injury. A videofluoroscopy study. *Neurogastroenterol Motil* **2012**, *24*, 414-419, e206, doi:10.1111/j.1365-2982.2011.01869.x.
46. Solazzo, A.; Monaco, L.; Del Vecchio, L.; Tamburrini, S.; Iacobellis, F.; Berritto, D.; Pizza, N.L.; Reginelli, A.; Di Martino, N.; Grassi, R. Investigation of compensatory postures with videofluoromanometry in dysphagia patients. *World J Gastroenterol* **2012**, *18*, 2973-2978, doi:10.3748/wjg.v18.i23.2973.
47. Cheng, I.; Hamad, A.; Sasegbon, A.; Hamdy, S. Advances in the Treatment of Dysphagia in Neurological Disorders: A Review of Current Evidence and Future Considerations. *Neuropsychiatr Dis Treat* **2022**, *18*, 2251-2263, doi:10.2147/ndt.S371624.
48. Calvo, I.; Sunday, K.L.; Macrae, P.; Humbert, I.A. Effects of chin-up posture on the sequence of swallowing events. *Head Neck* **2017**, *39*, 947-959, doi:10.1002/hed.24713.
49. Castell, J.A.; Castell, D.O.; Schultz, A.R.; Georgeson, S. Effect of head position on the dynamics of the upper esophageal sphincter and pharynx. *Dysphagia* **1993**, *8*, 1-6, doi:10.1007/bf01351470.
50. Logemann, J.A.; Kahrilas, P.J.; Kobara, M.; Vakil, N.B. The benefit of head rotation on pharyngoesophageal dysphagia. *Arch Phys Med Rehabil* **1989**, *70*, 767-771.
51. Ohmae, Y.; Ogura, M.; Kitahara, S.; Karaho, T.; Inouye, T. Effects of head rotation on pharyngeal function during normal swallow. *Ann Otol Rhinol Laryngol* **1998**, *107*, 344-348, doi:10.1177/000348949810700414.
52. Kim, C.K.; Ryu, J.S.; Song, S.H.; Koo, J.H.; Lee, K.D.; Park, H.S.; Oh, Y.; Min, K. Effects of Head Rotation and Head Tilt on Pharyngeal Pressure Events Using High Resolution Manometry. *Ann Rehabil Med* **2015**, *39*, 425-431, doi:10.5535/arm.2015.39.3.425.
53. Rasley, A.; Logemann, J.A.; Kahrilas, P.J.; Rademaker, A.W.; Pauloski, B.R.; Dodds, W.J. Prevention of barium aspiration during videofluoroscopic swallowing studies: value of change in posture. *AJR Am J Roentgenol* **1993**, *160*, 1005-1009, doi:10.2214/ajr.160.5.8470567.
54. Ertekin, C.; Keskin, A.; Kiylioglu, N.; Kirazli, Y.; On, A.Y.; Tarlaci, S.; Aydoğdu, I. The effect of head and neck positions on oropharyngeal swallowing: a clinical and electrophysiologic study. *Arch Phys Med Rehabil* **2001**, *82*, 1255-1260, doi:10.1053/apmr.2001.25156.
55. Lee, H.; Rho, H.; Cheon, H.-J.; Oh, S.M.; Kim, Y.-H.; Chang, W.H. Selection of Head Turn Side on Pharyngeal Dysphagia in Hemiplegic Stroke Patients: a Preliminary Study. *Brain Neurorehabil* **2018**, *11*.
56. Drake, W.; O'Donoghue, S.; Bartram, C.; Lindsay, J.; Greenwood, R. Eating in side-lying facilitates rehabilitation in neurogenic dysphagia. *Brain Inj* **1997**, *11*, 137-142, doi:10.1080/026990597123737.

57. Kaneoka, A.; Inokuchi, H.; Yamauchi, A.; Sakai, T.; Saito, Y.; Haga, N. Utility of side-lying posture in a patient with severe dysphagia secondary to oropharyngeal cancer surgery: A single case report. *Advances in Communication and Swallowing* **2022**, *25*, 29-36, doi:10.3233/acs-210039.
58. Lazarus, C.L. History of the Use and Impact of Compensatory Strategies in Management of Swallowing Disorders. *Dysphagia* **2017**, *32*, 3-10, doi:10.1007/s00455-016-9779-6.
